# Ampere-level furfurylamine electrosynthesis enabled by high-density atomic copper sites and a well-engineered electrolysis reactor

**DOI:** 10.1126/sciadv.aed7671

**Published:** 2026-05-08

**Authors:** Weiliang Zhou, Qihua Huo, Shuai Qi, Taiyu Liang, Jiao You, Chunyan Shang, Hengpan Yang, Xue Zhang, Lele Peng, Qi Hu, Chuanxin He

**Affiliations:** ^1^ College of Chemistry and Environmental Engineering, Shenzhen University, Shenzhen, Guangdong 518060, People’s Republic of China.; ^2^ Institute of Materials Research, Tsinghua Shenzhen International Graduate School Tsinghua University, Shenzhen, Guangdong 518055, People’s Republic of China.

## Abstract

Furfurylamine is an essential feedstock for diverse agrochemicals and pharmaceuticals; however, industrial-scale thermochemical furfurylamine production is plagued by high energy consumption and considerable greenhouse gas emissions. Here, we propose an electrosynthesis approach for converting biomass-derived furfural to furfurylamine, using nitrate as the nitrogen source under ambient conditions. We observe that single atomic copper (Cu-SA) sites favor the C─N coupling of nitrate-derived hydroxylamine and furfurals over hydroxylamine hydrogenation to ammonia (preferred by Cu subnanoclusters and nanoparticles), endowing Cu-SA with unique advantages in facilitating furfurylamine production. To enable industrial-scale furfurylamine production and suppress by-product formation, we design a single-pass continuous flow reactor with the separate liquid feeding of furfural and alkaline nitrate solutions and construct the highly dense Cu-SA sites. This integration achieves a large current of 2.3 amperes at a cell voltage of 1.9 volts, accompanied by an 84.53% single-pass conversion rate and 100% selectivity toward furfurylamine. Notably, techno-economic analysis demonstrates the profitability of our electrosynthesis furfurylamine route.

## Teaser

High-density atomic copper sites and an engineered reactor enable ampere-level electrosynthesis of furfurylamine.

## INTRODUCTION

**Fig. 1.**
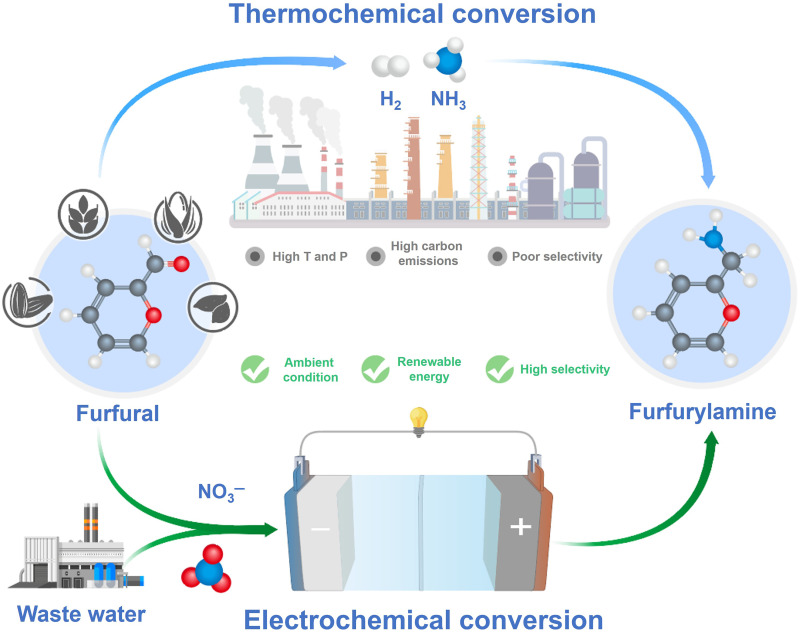
Comparison of the conventional thermochemical strategy and electrosynthesis strategy for producing furfurylamine. The former is conventional thermochemical strategy, and the latter uses biomass-derived furfural and NO_3_^−^ as reactants.

The reductive amination of aldehydes is a vital route toward synthesis of various primary amines (*1*–*3*). Because these amines have wide applications as essential feedstocks for agrochemicals, pharmaceuticals, and other value-added fine chemicals, this type of reaction has attracted ever increased attention (*4*–*8*). Moreover, a variety of aldehydes can be readily derived from lignocellulose, which offers excellent opportunities for the sustainable production of amines from biomass and thereby contributing to the reduction of carbon emissions (*9*–*11*). For example, the reductive amination of lignocellulose-derived furfural generates furfurylamine (*12*–*14*). Unfortunately, conventional amination reactions typically use high-pressure ammonia (NH_3_) and hydrogen (H_2_) as the nitrogen source and reductant, which are mainly produced from energy-consuming industrial routes (Fig. 1A, top half) (*15*, *16*). Moreover, the process also requires high reaction temperatures and precious metal-based catalysts, rendering such amine production route from the reaction of furfural/NH_3_/H_2_ costly and energy intensive (*17*–*19*). To develop an environmentally friendly route for producing primary amines with relatively low energy input, it is highly urgent to explore a low-cost nitrogen source alternative together with an energy-saving reaction process.

Electrosynthesis from earth-abundant and cheap reactants is an appealing strategy to produce value-added chemicals (*8*, *20*–*22*). For example, the nitrate (NO_3_^−^) reduction reaction (NO_3_^−^RR) not only addresses the environment issue of NO_3_^−^ but also efficiently produces NH_3_ (*23*–*27*). Recent achievements indicate that the hydroxylamine (*NH_2_OH) intermediates from the NO_3_^−^RR can react with formaldehyde intermediates (*HCHO) from carbon dioxide (CO_2_) reduction to generate methylamine during the electrochemical coreduction of NO_3_^−^/CO_2_ (*28*–*32*). With this in mind, we conceived that the *NH_2_OH may also react with furfural to produce furfurylamine, and, thus, the electrochemical coreduction of furfural and NO_3_^−^ under ambient conditions may be an energy- and cost-efficient route to produce amines (Fig. 1A, bottom half). The key to achieving this synthetic route is steering the coreduction of NO_3_^−^ and aldehydes toward C─N coupling and concurrently suppressing competitive reactions.

Herein, with the goal of reducing energy consumption and greenhouse gas emissions, we develop an electrosynthesis approach for furfurylamine via the coreduction of furfural and NO_3_^−^. We first screen electrocatalysts for the coreduction reaction from five metals, and metallic Cu is the optimum one. Furthermore, we endeavor to promote the key C─N coupling step in the coreduction by regulating the particle size of copper. It is observed that single atomic copper (Cu-SA) can promote the C─N coupling yet suppress the NH_3_ product generation, thereby achieving larger furfurylamine Faradaic efficiency (FE) than copper subnanoclusters (Cu-SNC) and Cu nanoparticles (Cu-NPs). Density functional theory (DFT) calculations reveal that Cu-SA sites can render C─N coupling step between the *NH_2_OH and *CHO groups of furfural thermodynamically more favorable than the hydrogenation of *NH_2_OH, thereby boosting the furfurylamine generation while concurrently suppressing NH_3_ by-products. In sharp contrast, Cu-SNC and Cu-NPs prefer the hydrogenation of *NH_2_OH over the coupling step, leading to the generation of NH_3_ by-products. To efficiently produce furfurylamine, we develop a Cu-SA electrocatalyst with an ultrahigh metal loading of 20 wt % and design a single-pass continuous flow reactor (SPCFR) electrolysis system. This system features the separate liquid feeding of furfural and alkaline nitrate solutions, which shortens the contact time between furfural and the alkaline medium to suppress the competitive self-reaction of furfural.

## RESULTS

### One-pot electrosynthesis of furfurylamine from the NO_3_^−^/furfural

**Fig. 2.**
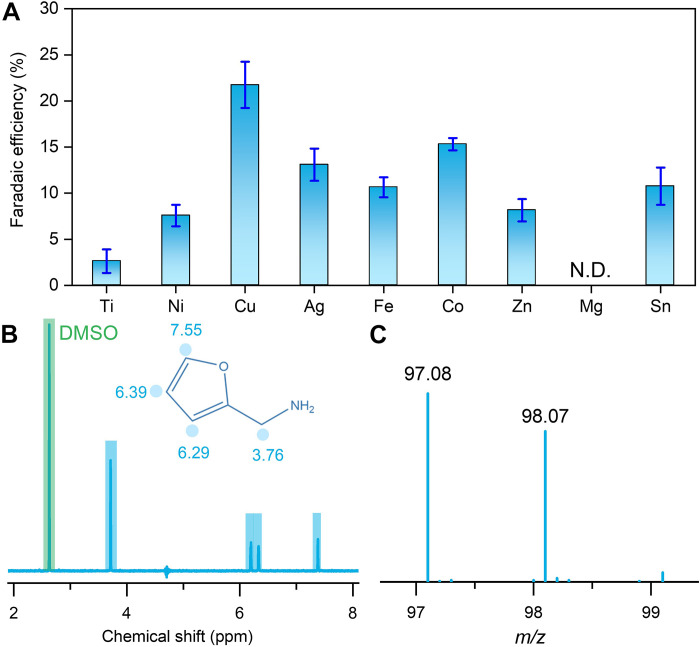
Catalyst screening and product detection. (**A**) Catalyst screening for furfurylamine electrosynthesis by using furfural and NO_3_^−^ as raw materials. N.D., not detected. (**B**) ^1^H NMR. (**C**) Liquid chromatography–mass spectrometry detection of furfurylamine in liquid products from the coreduction of furfural/NO_3_^−^. DMSO, dimethyl sulfoxide; *m*/*z*, mass/charge ratio; ppm, parts per million.

The coreduction of NO_3_^−^ and furfural is first performed in the electrolyte of 1 M KOH aqueous solution by using an H-cell. Nine different metal foils (i.e., Ni, Ti, Fe, Ag, Cu, Co, Zn, Mg, and Sn) are screened for the coreduction, and Cu foils display the largest current density and FE of furfurylamine (FE_FAM_) at −0.2 V versus reversible hydrogen electrode (RHE), indicating that Cu is an optimum metal for the coreduction (fig. S1 and Fig. 2A). The superior activity of Cu may be associated with the high activity for the NO_3_^−^RR and good capacity for activating aldehydes (*31*–*33*). The generated furfurylamine was detected by using ^1^H-nuclear magnetic resonance (^1^H NMR) (Fig. 2B and fig. S2), high-performance liquid chromatography (HPLC) spectroscopy (fig. S3), and liquid chromatography–mass spectrometry (Fig. 2C). Moreover, the liquid by-products (i.e., NO_2_^−^ and NH_4_^+^) were detected via ultraviolet-visible spectroscopy, and furfuryl alcohol was detected via ^1^H NMR and HPLC (figs. S4 to S9).

### Synthesis and structural characterization of various catalysts

**Fig. 3.**
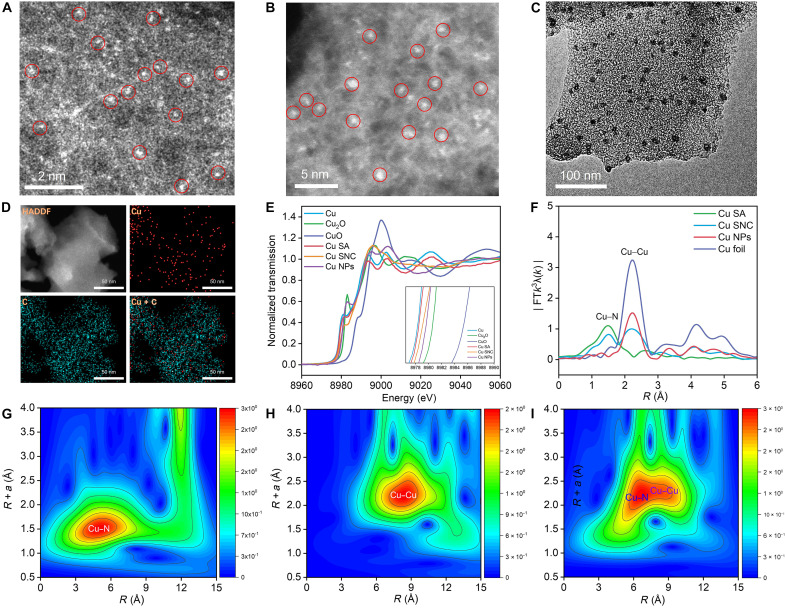
Characterization of the catalyst structure. High-angle annular dark-field scanning transmission electron microscopy (HAADF-STEM) images of (**A**) Cu-SA and (**B**) Cu-SNC. (**C**) Transmission electron microscopy (TEM) images of Cu-NPs. (**D**) EDS elements mapping of Cu-SA. (**E**) X-ray absorption near-edge structure (XANES) spectra at the Cu K-edge of Cu-SA, Cu-SNC, and Cu-NPs, as well as referenced Cu foil, Cu_2_O, and CuO. (**F**) The extended x-ray absorption fine structure (EXAFS) spectra at Cu K-edge of Cu-SA, Cu-SNC, and Cu-NPs, as well as referenced Cu foil. (**G** to **I**) Wavelet transforms for the *k*^3^-weighted EXAFS of Cu K-edge on Cu-SA, Cu-NPs, and Cu-SNC.

After determining that metallic Cu is a suitable metal for catalyzing the coreduction reaction, we sought to further enhance the catalytic performance of Cu by tuning the size of Cu. By using a confined strategy, three typical sizes of Cu-based electrocatalysts were synthesized, including Cu-SA, Cu-SNC, and Cu-NPs. Inductively coupled plasma mass spectrometry (ICP-MS) measurements indicate that the content of Cu-SA, Cu-SNC, and Cu-NPs is 1.15, 5.74, and 24.68%, respectively (table S1). Moreover, high-angle annular dark-field scanning transmission electron microscopy (HAADF-STEM) images of Cu-SA and Cu-SNC confirm the successful construction of atomic Cu sites and Cu cluster in diameter of ~1 nm on Cu-SA and Cu-SNC, respectively (Fig. 3, A to D, and figs. S10 and S11). Transmission electron microscopy (TEM) images of Cu-NPs and corresponding size-distribution histograms indicate that the average diameter of Cu-NPs is ~11 nm (Fig. 3C and fig. S12). X-ray absorption spectroscopy (XAS) was conducted to investigate the electronic state and coordination structure of Cu atoms in the above three samples. The Cu K-edge extended x-ray absorption near-edge structure (XANES) spectra manifest that the adsorption edges follow a sequence of Cu-SA > Cu-SNC > Cu-NPs (Fig. 3E), suggesting an increasing order of their valence states, in consistence with x-ray photoelectron spectroscopy (XPS) spectra of Cu 2p (figs. S13 and S14). This also implies that electronic structures of Cu can be well tuned by altering the particle size. The Fourier transform extended x-ray absorption fine structure (EXAFS) spectra of Cu-SA and Cu-NPs display a dominate peak at 1.47 and 2.24 Å corresponding to the Cu-N/C and Cu-Cu bonding, further confirming the generation of Cu-SA and Cu-NPs, respectively (Fig. 3, F and G). As for the Cu-SNC, two peaks are observed: one strong peak assigned to Cu─N/C bonding (Fig. 3, F and I) and one weak peak assigned to Cu─Cu bonding, and these characteristics are consistent with subnanometric metal clusters. Moreover, the fitting results of EXAFS for Cu-SA indicate that the coordination number of Cu-SA is ~4 (table S2), revealing that the structure of Cu-SA is CuN_4_. Overall, the above results demonstrate the successful synthesis of three Cu-based electrocatalysts with different diameters.

### Electrocatalytic activity evaluation of various catalysts toward coreduction of NO_3_^−^/furfural

**Fig. 4.**
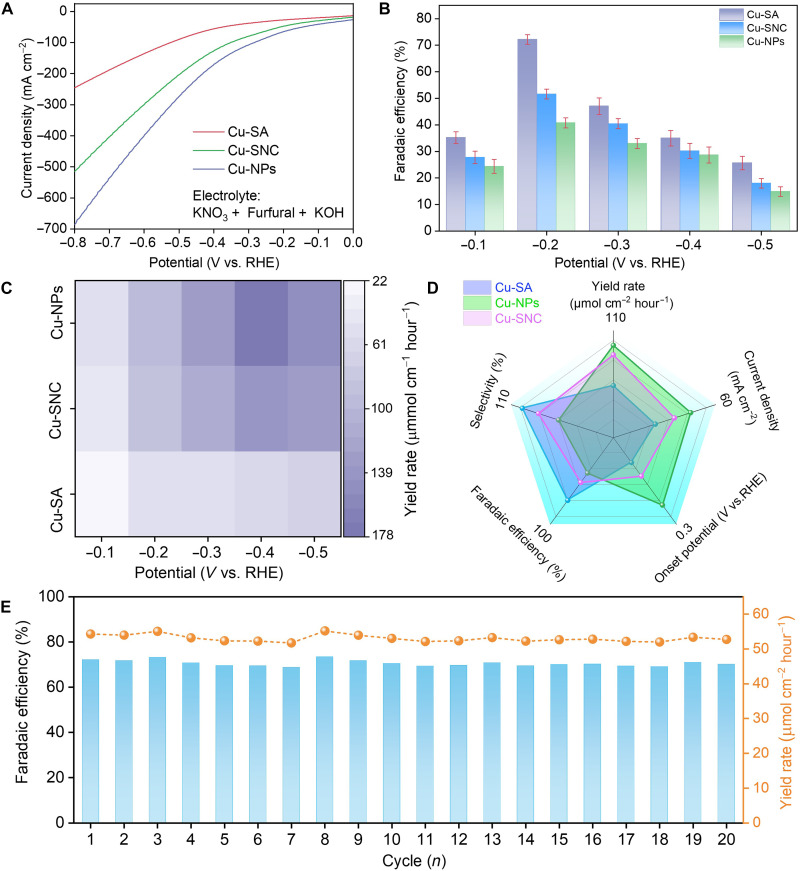
Electrochemical performance characterization. (**A**) LSV curves of Cu-SA, Cu-SNC, and Cu-NPs. (**B**) FE of furfurylamine on Cu-SA, Cu-SNC, and Cu-NPs at various potential for the coreduction of furfural/NO_3_^−^. (**C**) Yield rate of furfurylamine on Cu-SA, Cu-SNC, and Cu-NPs at various potential. (**D**) Comparisons between different Cu-based electrocatalysts based on various metrics, including selectivity, onset potential, current density, FE, and yield rate of furfurylamine. (**E**) Stability test of Cu-SA for the continuous 20 cycles of coreduction.

Cu-SA, Cu-SNC, and Cu-NPs were then used as electrocatalysts to perform the coreduction reaction in 1 M KOH aqueous solution containing 0.015 M furfural. The NO_3_^−^/furfural ratio was set to 3. As displayed in Fig. 4A and fig. S15, at the potential of −0.2 V versus the RHE, Cu-SA delivers a large current density of 10.94 mA cm^−2^, larger than the sole reduction of NO_3_^−^ (6.76 mA cm^−2^) and furfural (3.34 mA cm^−2^), indicating the possible occurrence of the coreduction for generating additional current densities. Furthermore, at a potential range from −0.1 to −0.5 V versus RHE, a maximum FE_FAM_ of 72.17% is achieved at −0.2 V versus RHE, along with 100% furfurylamine selectivity from furfural (Fig. 4B and figs. S16 and S17). In sharp contrast, at the same potential, Cu-SNC and Cu-NPs enable a small FE_FAM_ of 51.63 and 40.76%, respectively, suggesting that the Cu-SA promotes the generation of FAM. Furthermore, at −0.2 V versus RHE, the main by-products are NO_2_^−^ and NH_3_, and Cu-SA has smaller FEs of (NO_2_^−^ and NH_3_) than those of Cu-SNC and Cu-NPs, reflecting that the larger FE_FAM_ on Cu-SA is owing to the stronger ability to suppress these two by-product generation (figs. S18 to S20). As previously reported, the self-polymerization and disproportionation of furfural tend to occur in high concentration alkaline solution (*12*, *18*). To this end, we first examined furoic acid via ^1^H NMR spectroscopy, and the absence of characteristic peak for furoic acid (i.e., 7.6 parts per million) indicated the elimination of this by-product. Moreover, no polymeric by-products were observed in the liquid products, suggesting that the low furfural concentration (0.015 M) could suppress the Cannizzaro disproportionation and polymerization (fig. S21). The influence of potential on the coreduction was also investigated, indicating that a too negative potential could induce the generation of another by-product of furfuryl alcohol due to the direct reduction of furfural (figs. S22 and S23). Furthermore, we investigated the influence of furfural/NO_3_^−^ ratio on the generation of furfurylamine from the coreduction reaction, and the results indicate that the optimal ratio is 1:3 (fig. S24).

Although Cu-SA is highly selective for the C─N coupling reaction, the low metal loading (~1 wt %) greatly limits the reaction rate and current density. At −0.2 V versus RHE, Cu-SA delivers a current density of merely 27.09 mA cm^−2^, smaller than that of Cu-SNC (47.26 mA cm^−2^) and Cu-NPs (64.79 mA cm^−2^). As a result, at −0.2 V versus RHE, Cu-SA enables a smaller yield rate of FAM (54.29 μmol cm^−2^ hour^−1^) than Cu-NSC (85.33 μmol cm^−2^ hour^−1^) and Cu-NPs (94.95 μmol cm^−2^ hour^−1^) (Fig. 4, C and D, and fig. S25). Besides, we also investigated the influence of electrolyte pH on the FE_FAM_. As displayed in fig. S26, another by-product of furaldehyde oxime is detected in a neutral electrolyte, and FE_FAM_ reduces from 72.17 to 54.86% on Cu-SA when the electrolyte is changed from alkaline to neutral one. As previously reported, an alkaline electrolyte can markedly boost the water dissociation step relative to a neutral counterpart, and, thus, the generation of furaldehyde oxime may be attributed to the insufficient protons supply, which inhibits the further hydrogenation of furaldehyde oxime to furfurylamine (*34*). The electrochemical active surface area (ECSA) of various catalysts was measured through the double-layer capacitance (*C*_dl_) method, and the result indicated that the *C*_dl_ value of Cu-SA, Cu-SNC, and Cu-NPs was 6.43, 12.91, and 17.13 mF cm^−2^, respectively. To exclude the influence of ECSA on the electrocatalytic performance, we normalized the yield rate of furfurylamine by *C*_dl_ values, and Cu-SA (8.44 μmol mF^−1^ hour^−1^) displayed superior electrocatalytic performance than that of Cu-SNC (6.61 μmol mF^−1^ hour^−1^) and Cu-NPs (5.54 μmol mF^−1^ hour^−1^) (figs. S27 and S28). Moreover, Cu-SA displayed a smaller charge transfer resistance than the other two catalysts, which facilitates the coreduction process (fig. S29). Long-term stability is an important parameter to evaluate whether an electrocatalyst is suitable for practical applications. After 20 cycles of electrolysis, the FE_FAM_ and yield rate of FAM (Y_FAM_ )of Cu-SA remain nearly unchanged (Fig. 4E), and Cu-SA is still uniformly dispersed on the porous carbon without obvious aggregation, signifying the good stability of Cu-SA (fig. S30). As reported, Cu-SA dynamically transforms into Cu subnanoclusters at potentials below −0.6 V versus RHE during carbon dioxide (CO_2_) reduction (*35*). In our work, the operating potential of Cu-SA is −0.2 V versus RHE, which is insufficient to induce the dynamic reconstruction of Cu-SA; thus, Cu-SA retains its single atomic structure during the electrochemical coreduction of furfural/NO_3_^−^.

### Mechanism study of C─N coupling

**Fig. 5.**
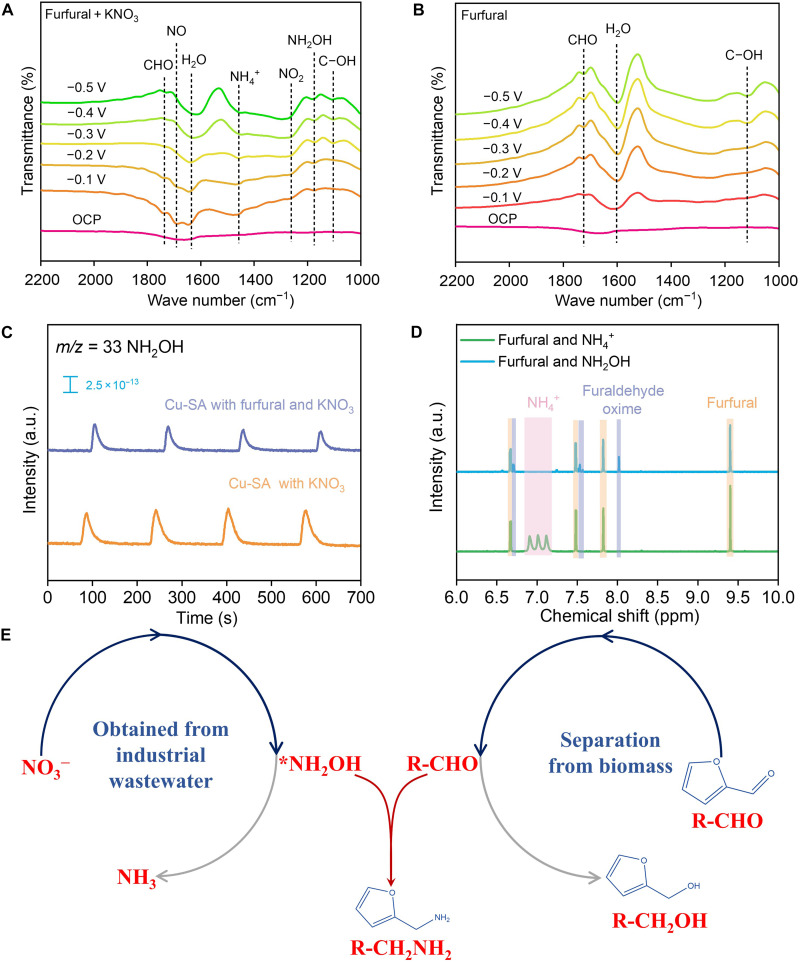
Mechanism exploration. (**A**) Operando attenuated total reflection Fourier transform infrared (ATR-FTIR) spectroscopy measurements on Cu-SA for the furfural/NO_3_^−^ coreduction under various potentials. OCP, Open Circuit Potential. (**B**) Operando ATR-FTIR spectroscopy measurements on Cu-SA for the sole furfural reduction under various potentials. (**C**) Operando differential electrochemical mass spectrometry (DEMS) results of NH_2_OH for Cu-SA and Cu-NP that catalyzed the sole NO_3_^−^ reduction and Cu-SA that catalyzed the furfural/NO_3_^−^ coreduction. (**D**) ^1^H NMR detection of furfurylamine from the spontaneous reaction of furfural and NH_2_OH without applied potential, whereas no furfurylamine is generated when NH_2_OH is changed to NH_4_^+^. (**E**) Schematic illustration of furfurylamine generation pathway on the surface of Cu-SA. a.u., arbitrary units; *m*/*z*, mass/charge ratio; ppm, parts per million.

To explore the C─N coupling pathway, operando attenuated total reflection surface-enhanced infrared absorption spectroscopy (ATR-SEIRAS) was performed to track the evolution of intermediates during the process of coreduction. At a reduction potential of −0.2 V versus RHE, ATR-SEIRAS displays signals of *CHO (1727 cm^−1^), *C─OH (1103 cm^−1^), *NO (1689 cm^−1^), *NO_2_ (1261 cm^−1^), *NH_2_OH (1176 cm^−1^), and NH_3_ (1457 cm^−1^) from the coreduction of furfural/NO_3_^−^ (Fig. 5A) (*36*–*38*). Control experiments with sole reduction of furfural and NO_3_^−^ suggests that the *CHO signal originates from furfural, while the *NO_3_, *NO_2_, *NH_2_OH, and *NH_3_ originate from NO_3_^−^ (Fig. 5B and fig. S31). It should be noted that *NH_2_OH is the most nucleophilic species in these N-containing intermediates, implying that the *NH_2_OH and *CHO of furfural may be precursors for the C─N coupling. Moreover, the peak of *C─OH could be referred to as the peak of C─O bond in furfuryl alcohol, and corresponding signals appeared from −0.3 V versus RHE, further confirming that furfuryl alcohol by-products could not be generate at −0.2 V versus RHE. To rule out the contribution of NH_3_ for the C─N coupling, we replaced the reactant of NO_3_^−^ by NH_4_^+^ and NH_3_, and no furfurylamine is detected in the liquid product, indicating that NH_3_ is not involved in the C─N coupling. Notably, the utilization of NO_2_^−^ and NH_2_OH as N resources enables furfurylamine generation, suggesting that *NH_2_OH may be involved in the C─N coupling (table S3). The C─N coupling pathway was further identified by using online differential electrochemical mass spectrometry (DEMS). First, DEMS results of the sole NO_3_^−^ reduction on Cu-SA display an obvious signal of mass/charge ratio of 33 corresponding to *NH_2_OH (fig. S32). Second, the intensity of *NH_2_OH is significantly weakened for the NO_3_^−^/furfural coreduction with respect to the sole NO_3_^−^ reduction, and the weakened intensity can be attributed to the consumed *NH_2_OH originated from the C─N coupling reaction, further confirming that *NH_2_OH is involved in the C─N coupling (Fig. 5C). To gain direct evidence, we mixed 10 mM furfural and 10 mM NH_2_OH at room temperature (no applied potential) and collected ^1^H NMR spectra of such mixture within 30 min. The results demonstrate that NH_2_OH reacts spontaneously with furfural to generate furaldehyde oxime at a rate of 0.33 mM min^−1^ (fig. S33). In sharp contrast, no furaldehyde oxime is generated when NH_2_OH is changed to NH_3_, undoubtedly verifying that NH_3_ is not involved in the C─N coupling (Fig. 5D and fig. S34). On basis of all above results, we deduce the reaction pathway of furfural/ NO_3_^−^ coreduction including the first reduction of NO_3_^−^ to generate *NH_2_OH, followed by the C─N coupling step to yield furaldehyde oxime, which is further reduced to furfurylamine (Fig. 5E).

### Theoretical calculation

**Fig. 6.**
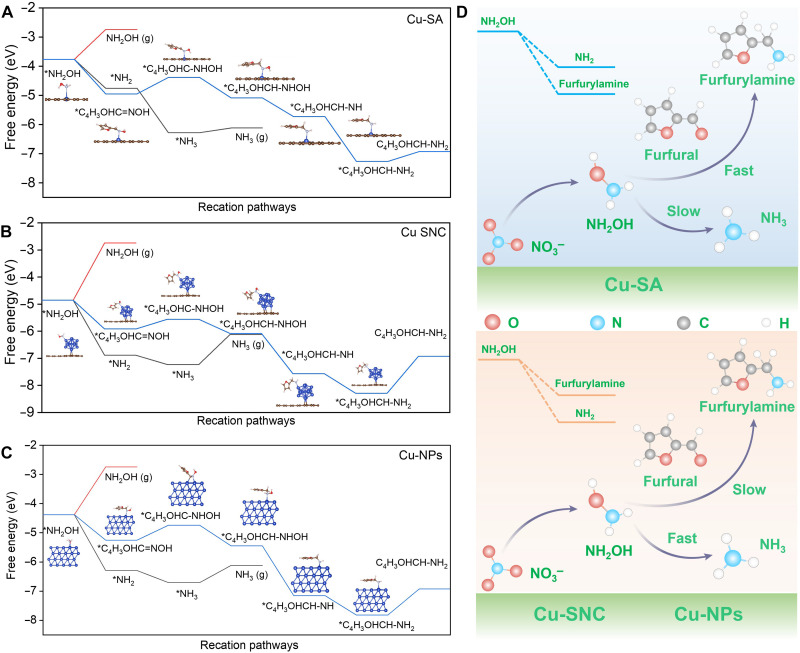
DFT calculations. DFT-calculated Gibbs free energy profiles of the furfural/ *NH_2_OH intermediates coreduction to generate furfurylamine over (**A**) Cu-SA (**B**), Cu-SNC, and (**C**) Cu-NPs. (**D**) Schematic illustration in which the Cu-SA sites favor the furfurylamine generation and suppress NH_3_ by-product generation, whereas Cu-SNC and Cu-NP sites instead favor NH_3_ by-product generation over furfurylamine.

DFT calculations were conducted to understand origins behind the superior performance of Cu-SA for the C─N coupling toward furfurylamine. In the coreduction of furfural/NO_3_^−^, once *NH_2_OH intermediates are generated, three reaction pathways are possible: (i) desorption of *NH_2_OH to form NH_2_OH; (ii) reaction with furfural to produce furaldehyde oxime; and (iii) hydrogenation of *NH_2_OH to *NH_2_, followed by further conversion to NH_3_. On the surfaces of Cu-SA, Cu-SNC, and Cu-NPs, only path (i) proceeds as an uphill process, whereas paths (ii) and (iii) both occur as downhill processes, suggesting path (i) is less likely to take place on these catalyst surfaces. As for paths (ii) and (iii), Cu-SA renders path (ii) (*NH_2_OH → *C_4_H_3_OHC═NOH, −1.18 eV) more thermodynamically favorable than path (iii) (*NH_2_OH → *NH_2_, −0.99 eV). In sharp contrast, on both Cu-SNC and Cu-NPs, path (iii) is more favorable instead of path (ii). In other words, Cu-SNC and Cu-NPs facilitate the direct hydrogenation of *NH_2_OH to *NH_2_ and subsequent *NH_3_ rather than the coupling with *CHO, leading to a relatively low FE_FAM_ and high FE_NH3_ (Fig. 6, A to C). Therefore, the fate of *NH_2_OH dictates the branching between furfurylamine and NH_3_, and Cu-SA sites enable a stronger preference toward the coupling of *NH_2_OH and *CHO, thereby achieving a significantly enhanced FE_FAM_ (Fig. 6D). Note that the calculation of the Gibbs free energy change of a reaction is only applicable for evaluating the spontaneity trend of the reaction pathway over a catalyst. Accordingly, the fact that the Gibbs free energy required for the hydrogenation of *NH_2_OH is lower than that for C─N coupling over Cu-SNC and Cu-NPs does not imply that the yield of NH_3_ is higher than that of furfurylamine. In this regard, our DFT calculations only demonstrate that the Cu-SA has a superior ability to suppress the hydrogenation of *NH_2_OH to generate NH_3_ by-products than Cu-SNC and Cu-NPs, consistent with our experiment results. Furthermore, in the overall coreduction process of *NH_2_OH and furfural to form furfurylamine, the desorption of furfurylamine is the potential-determining step (PDS) over all the three catalysts. Notably, Cu-SA requires a lower energy of 0.34 eV for the PDS compared to Cu-SNC (1.37 eV) and Cu-NPs (0.90 eV), thereby promoting the generation of furfurylamine over Cu-SA (figs. S35 to S40). Besides, we also investigated the activity other single atomic metal sites (i.e., Co-SA, Ni-SA, and Zn-SA). As displayed in fig. S41, DFT calculations indicate that all the three electrocatalysts prefer *NH_2_OH hydrogenation to *NH_2_ instead of its coupling with *CHO, leading to the inferior performance than Cu-SA for the coreduction toward furfurylamine. To experimentally verify it, we synthesized Co-SA, Ni-SA, and Zn-SA. As expected, Cu-SA displays a higher FE of furfurylamine than the other three single atomic metal catalysts, further confirming the superior performance of Cu-SA in furfurylamine generation from the coreduction of furfural/NO_3_^−^ (figs. S42 to S46).

### Substrate expansion, scalable furfurylamine electrosynthesis, and techno-economic analysis

To investigate whether the electrosynthesis route is generalized for various primary amines generation, we expanded the substrates to 5-methylfurfural, benzaldehyde, 3-phenylpropionaldehyde, anisaldehyde, *p*-methylbenzaldehyde, and *p*-chlorobenzaldehyde (figs. S47 to S58). As displayed in table S4, at −0.2 V versus RHE, more than 34.18% FEs along with a yield rate of 23.07 μmol hour^−1^ cm^−2^ can be achieved toward six different primary amines, suggesting that the electrosynthesis route via the coreduction of aldehyde and NO_3_^−^ can be extended to various primary amine production.

**Fig. 7.**
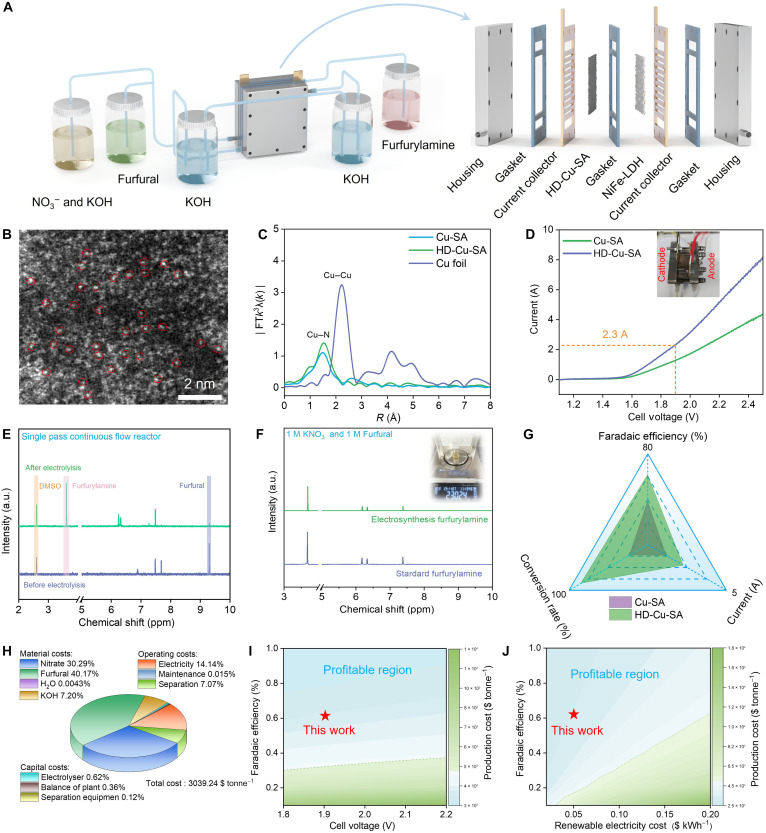
Large-scale electrosynthesis of furfurylamine. (**A**) Schematic illustration of the SPCFR system. (**B**) HAADF-STEM images of HD-Cu-SA. (**C**) The EXAFS spectra at Cu K-edge of Cu-SA, HD-Cu-SA, and referenced Cu foil. (**D**) LSV curves of Cu-SA and HD-Cu-SA acquired via the SPCFR system, and the inset is the optical photograph of SPCFR system. (**E**) ^1^H NMR spectra of solution before and after the 5-hour coreduction reaction via the SPCFR. (**F**) ^1^H NMR spectra of furfurylamine purified from the electrolyte of coreduction reaction and standard furfurylamine, with the inset showing pure furfurylamine isolated from the 5-hour continuous electrolysis electrolyte. a.u., arbitrary units; DMSO, dimethyl sulfoxide; ppm, parts per million. (**G**) Comparison of current, furfural conversion rate, and furfurylamine selectivity on Cu-SA and HD-Cu-SA. (**H**) Total cost of furfurylamine electrosynthesis under large-scale operating conditions, with the electricity price assumed to 0.05 $ kWh^−1^. (**I**) Sensitivity analysis of costs with respect to cell voltage and FE. (**J**) Impact of FE and renewable electricity cost on furfurylamine electrosynthesis cost.

As discussed above, the low metal loading of Cu-SA greatly limits the current density and furfurylamine yield rate (Fig. 4A). To address this issue, we synthesized highly dense Cu-SA (denoted as HD-Cu-SA) with an ultrahigh loading of 20 wt % using graphene quantum dots as supports. Corresponding HAADF-STEM images and EXAFS results confirm the successful synthesis of HD-Cu-SA (Fig. 7, B and C). Then, we attempt to synthesize gram-scale high-purity furfurylamine using concentrated furfural and NO_3_^−^ as reactants, with HD-Cu-SA serving as the electrocatalyst. However, a high furfural concentration (i.e., 0.3 M) required for large-scale furfurylamine production may promote the side reactions of Cannizzaro disproportionation or polymerization (*10*). In a convenient flow cell with the cointroduction of 0.3 M furfural and 1 M KOH, an obvious ^1^H NMR peak for furoic acid was observed, as well as the appearance of suspended furfural-derived polymers in the liquid products, suggesting that a high furfural concentration inevitably induced the by-product generation (fig. S59). To address this issue, we designed a SPCFR electrolysis system instead of conventional flow reactor system. In the SPCFR system, furfural and the mixed solution containing NO_3_^−^ and KOH were separately introduced into the cathode tank (Fig. 7A). Specifically, the mixed solution (NO_3_^−^/KOH) was fed at the front, while furfural was supplied at the rear. This configuration shortens the contact time between furfural and the alkaline solution, thereby suppressing the occurrence of side reactions (Fig. 7A and fig. S60). Notably, by using a large-area electrode (2 cm by 2 cm), HD-Cu-SA delivers an ultrahigh current of 2.3 A at a cell voltage of 1.9 V via the SPCFR system, and the current is 1.8-fold larger than Cu-SA, suggesting that the construction of highly dense Cu-SA sites efficiently enhances the current of the coreduction reaction (Fig. 7, D and G). At a cell voltage of 1.9 V, HD-Cu-SA achieves a maximum FE of 61.37% for furfurylamine, along with an 84.53% single-pass conversion rate and 100% selectivity toward furfurylamine (fig. S61). The 100% furfurylamine selectivity suggests that the SPCFR system can effectively inhibit the self-polymerization and disproportionation of furfural. Furthermore, 2.30 g of pure furfurylamine was obtained after 5 hours of electrolysis via simple extraction and evaporation of the organic solvent, underscoring the great potential for large-scale furfurylamine production (Fig. 7F). Besides, we comprehensively characterized the postreaction Cu-SA catalyst after 100 hours of continuous electrolysis at a cell voltage of 1.9 V via HAADF-STEM, XPS, EXAFS, and ICP-MS. The results demonstrate that its single-atomic dispersion, mass loading, coordination structure, and valence state remained nearly unchanged, thus validating the outstanding stability of Cu-SA in the coreduction reaction (figs. S62 and S63 and table S5).

A techno-economic analysis was conducted to explore the economic potential of furfurylamine electrosynthesis via the electrocatalytic coreduction of NO_3_^−^ and furfural as feedstock (*39*–*41*) (for details, see the Supplementary Materials). As depicted in Fig. 7H, with an assumed electricity price of 0.05 $ kilowatt-hour (kWh)^−1^, the large-scale electrosynthesis cost of furfurylamine is 3039.24 $ tonne^−1^, 1.61-fold lower than the commercial price (4908.84 $ tonne^−1^). Moreover, the influences of various operating conditions and renewable electricity prices on furfurylamine production costs were considered. The results show that furfurylamine electrosynthesis is profitable under our conditions, further verifying the feasibility and profitability of the renewable energy-driven coreduction of NO_3_^−^ and furfural to furfurylamine (Fig. 7, I and J).

## DISCUSSION

In summary, we have demonstrated a low-energy input and environment-friendly electrosynthesis strategy for furfurylamine by using furfural and NO_3_^−^ as sources. After screening various metals and optimizing the metal particle size, Cu-SA emerges as an optimum electrocatalyst for the C─N coupling reaction. DFT calculation suggest that the fate of *NH_2_OH determines branching to furfurylamine versus NH_3_ by-products, and Cu-SA enables a stronger preference toward the coupling of *NH_2_OH and CHO* groups of furfural than the hydrogenation of *NH_2_OH, thereby achieving significantly enhanced FE_FAM_. Notably, by integrating the high-density Cu-SA electrocatalyst with a well-engineered SPCFR electrolysis system, we achieve an industrial current of 2.3 A for the coreduction reaction along with obtaining 2.30 g of high-purity furfurylamine over 5 hours. Techno-economic analysis demonstrates the great potential of electrolysis route for the industrial furfurylamine production on basis of the production cost. We envision that the above findings can inspire further practical application of electrosynthesis route for various important chemicals production.

## MATERIALS AND METHODS

### Methods

#### Synthesis of N-doped porous carbon

A porous carbon nanomaterial with a large Brunauer-Emmett-Teller surface area of 2524 m^2^ g^−1^ and abundant micropores was purchased from Nanjing XFNANO Materials Tech Co. Ltd. For the synthesis of N-doped porous carbon, 0.3 g of the above porous carbon was mixed with 1.5 g of melamine, followed by calcination at 800°C in Ar for 2 hours with a ramping rate of 5°C min^−1^.

#### Synthesis of Cu-SA, Cu-SNC, and Cu-NPs

Cu-SA was obtained via freeze drying and subsequently controllable calcination. Specifically, 100 mg of the N-doped porous carbon above synthesized and 0.75 mM CuCl_2_ were dispersed in 20 ml of H_2_O to form a suspension via ultrasonic bath. The suspension was then stirred for 24 hours, and the resulting black powder was collected by freeze-drying. Last, Cu-SA was obtained by the black powder calcined at 300°C in Ar for 60 min. The synthetic processes of Cu-SNC and Cu-NPs were similar to that of Cu-SA, except that the amounts of CuCl_2_ added were 2.25 and 9.5 mM, respectively, and the annealing temperature was served as 300° and 500°C in 5% H_2_/Ar for 60 min.

#### Synthesis of HD-Cu-SA

HD-Cu-SA was synthesized according to previous literatures by using amino-modified graphene quantum dot (GQD-NH_2_; purchased from XFNANO) as the support (*42*). In a typical process, 2 mM CuCl_2_ was mixed with 80 ml of GQD-NH_2_ solution (1 mg ml^−1^) to form a homogeneous solution, followed by sonication in ice water for 15 min. Then, the above solution was freeze dried and mixed with urea with a mass ratio of 1to10. Last, HD-Cu-SA was obtained through annealing for 400°C for 1 hour in a tube furnace under the atmosphere of Ar.

#### Material characterization

Images of TEM, HAADF-STEM, and elemental mapping were collected on a JEOL JEM-F200 (2020) sorptometer. X-ray diffraction patterns were recorded on a D8ADVANCE diffractometer with Cu Kα radiation. The chemical state of Cu-based catalysts was investigated by using a Thermo VG Scientific ESCALAB 250 x-ray photoelectron spectrometer (Thermo Electron, UK). XAS including XANES and EXAFS of Cu-based catalysts was obtained at Spring-8 14b2 in Japan, in which a pair of channel-cut Si (111) crystals was used in the monochromator.

#### Electrochemical coreduction of NO_3_^−^/furfural measurements

Electrochemical measurements were performed in typical three-electrode system, which included carbon paper loaded with electrocatalysts (1 cm by 1 cm), an Hg/HgO electrode, and a piece of Pt foil as the working electrode, reference electrode, and counter electrode, respectively. An anion exchange membrane (Sustainion X37-50 Grade RT) was used to separate the cathode and anode in a typical H-cell, with 0.045 M KNO_3_, 0.015 M furfural, and 1 M KOH severing as the electrolyte. The catalyst ink was prepared by dispersing 4 mg of electrocatalyst in a mixed solution of 950 μl of ethanol and 50 μl of Nafion (5 wt % aqueous solution) with sonication for 30 min. Then, 100 μl of the ink was loaded onto the carbon paper as the working electrode. All Linear Sweep Voltammetry (LSV) curves were collected without IR compensation, and all the potentials were converted into the RHE scale using the following equation

$$E_{\text{RHE}}=E_{\text{Hg}/\text{HgO}}+0.0977+0.0591\times \text{pH}$$

#### Electrochemical coreduction of NO_3_^−^/furfural in the SPCFR system

The SPCFR system was constructed based on a zero-gap electrolyzer. The cathode compartment and anode compartment were partitioned by an anion exchange membrane, where the cathode and anode are the HD-Cu-SA and NiFe LDH with electrode geometric area of 4 cm^−2^. Furfural (1 M) and the mixed aqueous solution containing 1 M NO_3_^−^ and 1 M KOH were separately fed into the cathode compartment at flow rates of 0.1 and 0.3 ml min^−1^, respectively, while an aqueous KOH solution (1 M) is served as the electrolyte of anode. Specifically, the mixed solution (NO_3_^−^/KOH) was fed at the front, while furfural was supplied at the rear. The catalyst ink was prepared by dispersing 20 mg of electrocatalyst in a mixed solution of 3.8 ml of ethanol and 0.2 ml of Nafion (5 wt % aqueous solution) with sonication for 60 min. Then, 4 ml of the ink was loaded onto the carbon paper as the working electrode.

#### Calculation of FE and selectivity

The FE of furfurylamine was calculated by the following equation

FE(%)=(10×V×c×F)/(97.12×Q)×100%

The selectivity of glycine was calculated by the following equation

$$\text{Selectivity}=n_{\text{furfurylamine}}/\left(n_{\text{furfurylamine}}+n_{\text{furfural}}+n_{\text{furaldehyde oxime}}\right)$$

where *n*_furfurylamine_ and *n*_furaldehyde oxime_ are the generation of furfurylamine and furaldehyde oxime from coreduction of NO_3_^−^/furfural.

## Supplementary Materials

### This PDF file includes:

Sections S1 to S8 Figs. S1 to S63 Tables S1 to S5 References
