# Supplementary material for: Ampere-level furfurylamine electrosynthesis enabled by high-density atomic copper sites and a well-engineered electrolysis reactor
Source: Sci Adv. 2026 May 8;12(19):eaed7671. doi: 10.1126/sciadv.aed7671 (PMC13155300; doi:10.1126/sciadv.aed7671)
Supplement: Supplementary file 1 — Sections S1 to S8 Figs. S1 to S63 Tables S1 to S5 References [file sciadv.aed7671_sm.pdf]

Science Advances
AAAS

Supplementary Materials for

Ampere-level furfurylamine electrosynthesis enabled by high-density atomic copper sites and a well-engineered electrolysis reactor

Weiliang Zhou *et al.*

Corresponding author: Qi Hu, hq2016@szu.edu.cn; Chuanxin He, hecx@szu.edu.cn

Sci. Adv. **12**, eaed7671 (2026)
DOI: 10.1126/sciadv.aed7671

This PDF file includes:

Sections S1 to S8
Figs. S1 to S63
Tables S1 to S5
References

Supplementary Sections S1

The determination of ammonia

The method of indophenol blue was used to determine the concentration of NH_4^+. In a typical process, 2 mL colorant (the solution of 1 M sodium hydroxide, 5% salicylic acid, and 5% sodium citrate), 1.0 mL of 0.05 M NaClO and 0.2 mL of 1% mass fraction of sodium nitroferricyanide aqueous solution were dissolved in 2 mL of the obtained electrolyte from the co-reduction. Then, the obtained solution was mixed evenly and reacted in the dark for 2 hours. Finally, the absorbance of each sample at the wavelength of ~662 nm was measured. The standard curve for the quantification of ammonia was displayed in **Fig. S6** *(25)*.

Supplementary Sections S2

The determination of nitrite

The concentration of NO_2^- was determined via ultraviolet-visible (UV-vis) spectroscopy. In a typical process, N-(1-naphthyl) ethylenediamine dihydrochloride (5 mg) and sulfonic acid (0.5 g) were dissolved in 100 mL of deionized water to form a homogeneous solution. Then, 5 mL of acetic acid was added into the above solution, followed by the addition of 1 mL of the obtained electrolyte from the co-reduction and 4 mL of colorant form a homogeneous solution. Finally, the absorbance of the solution was detected at the wavelength of ~510 nm, and the standard curve of nitrite quantification is shown in **Fig. S7** *(24)*.

Supplementary Sections S3

The determination of furfurylamine

Furfurylamine was first quantified by Nuclear Magnetic Resonance Hydrogen Spectrum (^1H NMR). D_2O and DMSO served as the deuterated reagent and internal standard, respectively. 400 μL of the obtained electrolyte from the co-reduction was mixed with 100 μL of D_2O and 100 μL of DMSO, and the mixture was analyzed by ^1H

NMR. The ^{1}H NMR results presented in this work were the accumulated results of 32 scans using a 500 MHz NMR instrument (Bruker) equipped with an ultra-low-temperature probe. The standard curve of ^{1}H NMR for the quantification of furfurylamine was shown in **Fig. S2.**

To accurately quantify the content of furfurylamine, high-performance liquid chromatography (HPLC, Agilent 1260 Infinity II Prime) was also employed. HPLC was performed on a AQ C18 5 μm column (250 mm × 4.6 mm). The corresponding mobile phase, flow rate, and detection wavelength were 0.02 mol L^{-1} potassium dihydrogen phosphate and methanol (40:60), 0.8 mL min^{-1}, and 220 nm, respectively. The HPLC standard curve for quantifying furfurylamine was shown in **Fig. S3**. Additionally, the product was further confirmed to be furfurylamine by liquid chromatography-mass spectrometry (LC-MS). In a typical process, furfurylamine was separated from the electrolyte and detected by LC-MS

Supplementary Sections S4

Purification of furfurylamine

Firstly, the pH of electrolyte after electrolysis were adjusted to ~2.5 with 1 M H_2SO_4. Then, the ether with 1.5-fold the volume of electrolyte as extractant was added to the acidified electrolyte. After full oscillation and left stand, the acidified electrolyte was stratified, and the furfural was completely removed after repeated the extraction for 3 times. Next, the pH of separated aqueous were adjust the ~11 with 2 M KOH, and then the similar operation as the removal of furfural, the organic phase was separated and collected by the extraction of ether. Moreover, Na_2SO_4 served as desiccant to remove water in organic phase. Finally, pure furfuramine was obtained by evaporating the ether at 60 °C.

Supplementary Sections S5

Techno-economic Analysis (TEA)

The techno-economic analysis was conducted to explore the economic potential for the

electrosynthesis of furfurylamine through the electrocatalytic process utilizing NO_3^- and Furfural as feedstock. According to our experimental results, we investigated the impact of various operating condition and renewable electricity price on the cost of producing furfurylamine. The specific assumptions for the calculation of the cost for production furfurylamine are listed as follows *(39-41)*:

1. Operating condition: Current density is 2.3 A. Voltage is 1.9 V. Faradaic efficiency is 61%. Daily production is 50,000 kg of furfurylamine.
2. The price of H_2O is 0.7 \$ ton^{-1}. The price of furfural is 1234.22 \$ ton^{-1}. The price of nitrite is 350.63 \$ ton^{-1}. The price of sodium hydroxide was 378.68 \$ ton^{-1}. The price of hydrogen was 0.0015 \$ ton^{-1}. The price of furfurylamine is 4207.57 \$ ton^{-1}, all the cost of reactants come from the https://www.100ppi.com and the published literature *(39-41)*. The stack cost is set as 275.55 \$ kW^{-1}.
3. The anticipated service life of the electrolyzer was 15 years and the working time per year was 350 days.
4. The electrolyzer cost per area was calculated as follows:

$$\text{Electrolyzer cost per area} = 275.55\ \$kW^{-1} \times \frac{0.575\ A}{cm^{-2}} \times 1.9\ V \times \frac{10^4 cm^2}{m^2} \times \frac{kW}{1000W} = 3010.38\ \$m^{-2}$$

5. The cost of Balance of Plant was set as 58% of the cost of the electrolyzer.
6. The maintenance cost was set as 2.5% of the electrolyzer cost.

7. The cost of separation equipment is assumed to be 20% of the electrolyzer cost.
8. The relevant paraments are calculated as:

$$\text{Total current} = \frac{50000kg}{day} \times \frac{day}{86400s} \times \frac{10^3 g}{kg} \times \frac{mol}{97.12g} \times 10e^- \times \frac{96485C}{mol} \times \frac{1}{0.61} = 9424917.434\ A$$

$$\text{Electrolyzer Area} = 9424917.434\ A \times \frac{cm^2}{0.575\ A} \times \frac{m^2}{10^4 cm^2} = 1639.116\ m^2$$

$$\text{Power} = 1.9\ V \times 9424917.43387\ A \times \frac{W}{10^6 MW} = 17.907\ MW$$

The cost calculations for electrochemical synthesis of furfurylamine using nitrite and furfural are as follows:

Capital Cost:

1. Electrolyzer cost:

$$\text{Electrolyzer Cost per ton furfurylamine} = 2265.710\ \$m^{-2} \times 1639.116\ m^2 \times \frac{1}{15year} \times \frac{year}{350day} \times \frac{day}{50\ ton} = 18.797\ \$\ ton^{-1}$$

2. Balance of Plant:

$$\text{Balance of Plant per ton furfurylamine} = 18.797\$\ ton^{-1} \times 0.58 = 10.902\ \$\ ton^{-1}$$

3. Separation Equipment cost:

$$\text{Separation Equipment cost per ton furfurylamine} = 18.797\ \$\ ton^{-1} \times 0.2 = 3.759\ \$\ ton^{-1}$$

Operating Cost:

1. Electricity cost:

$$\text{Electricity cost} = 17.907\ MW \times \frac{1000kW}{MW} \times 24 \frac{h}{day} \times 0.05 \times \frac{\$}{kWh} \times \frac{day}{50\ ton} = 429.776\ \$\ ton^{-1}$$

2. Maintenance:

$$\text{Maintenance cost} = 14.1477\ \$\ ton^{-1} \times 0.025 = 0.470\ \$\ ton^{-1}$$

3. Separation:

$$\text{Separation cost}=646.926\ \$\ \text{ton}^{-1}\times 0.5=214.888\ \$\ \text{ton}^{-1}$$

Material Cost:

According to the experimental results, the total cost for the production of one ton of furfurylamine

$$\text{Material cost}=920.732\ \$\ \text{ton}^{-1}+1221.005\ \$\ \text{ton}^{-1}+0.130\ \$\ \text{ton}^{-1}+218.779\ \$\ \text{ton}^{-1}=2360.646\ \$\ \text{ton}^{-1}$$

Total Cost:

$$\text{Total cost}=18.797\ \$\ \text{ton}^{-1}+10.902\ \$\ \text{ton}^{-1}+3.759\ \$\ \text{ton}^{-1}+429.776\ \$\ \text{ton}^{-1}+0.470\ \$\ \text{ton}^{-1}+214.888\ \$\ \text{ton}^{-1}+2360.646\ \$\ \text{ton}^{-1}=3039.239\ \$\ \text{ton}^{-1}$$

Supplementary Sections S6

Operando DEMS measurements

The reaction intermediates were detected by DEMS (QAS100). For the co-reduction of furfural and NO_3^-, the electrolyte consisted of 0.045 M KNO_3, 0.015 M furfural, and 1 M KOH solution. Ar gas was bubbled into the electrolyte both before and during DEMS measurement. Signals were collected under electrolysis conditions at the potential of −0.2 V vs. RHE.

Supplementary Sections S7

Electrochemical operando ATR-FTIR measurements

The Si prisms were polished with an Al_2O_3 suspension and sequentially ultrasonically cleaned in a bath of acetone and deionized water before depositing a certain thickness of Au onto the surface of the Si prism. The prepared catalyst ink was loaded onto the above prepared Au film to prepare the working electrode. ATR-SERAS measurements were performed in a two-compartment spectroscopic electrochemical cell containing three electrodes, including a working electrode, a standard Ag/AgCl electrode as a reference electrode, and a platinum wire as a counter electrode, respectively. All ATR-SERAS spectra were acquired using a Fourier transform infrared spectrophotometer (FT-IR, Nicolet Is50 Thermo Fischer Scientific) equipped with a mercury cadmium telluride (MCT) detector. Electrochemical tests were performed in 0.045 M KNO_3, 0.015 M furfural and 1 M KOH solution and controlled by a CHI electrochemical

workstation (CHI760E). In a typical test, the working electrode was initially activated by running CV cycles between 0 and −0.6 V vs. RHE at a scan rate of 0.05 V s^{-1} until the system was stabilized. Then, the spectrum was collected under the different electrolytic conditions.

Supplementary Sections S8

Theoretical calculation method

All the calculations are performed in the framework of the density functional theory with the projector augmented plane-wave method, as implemented in the Vienna ab initio simulation package *(43)*. In this work, all calculations were carried out based on the VASP code. The generalzied gradient approximation proposed by Perdew, Burke, and Ernzerhof is selected for the exchange-correlation potential *(44)*. The energy cutoff was set to 400 eV. The 2*2*1 Monk-horst k-points grids was set during the calculations. The vacuum region was set to be 15 Å in z direction to prevent the interaction between two adjacent surfaces. The energy convergence was set to 10^{-5} eV. A geometry optimization was considered convergent when the energy change was smaller than 0.02 eV $Å^{-1}$. VASPsol provides support for Perform solvation calculations by VASPsol to consider the implicit solvation model. The bottom two layers were fixed during the calculation process. The Gibbs free energy change of each elementary step can be calculated by $\Delta G = \Delta E + \Delta E_{ZPE} - T\Delta S$. ΔE is the reaction energy, ΔE_{ZPE} is zero-point energies, T is the temperature (298.15 K), ΔS is the difference in entropy from vibrational frequency calculations. The entropies of gas phase N_2 and H_2 are obtained from the NIST database with standard condition *(45)*.

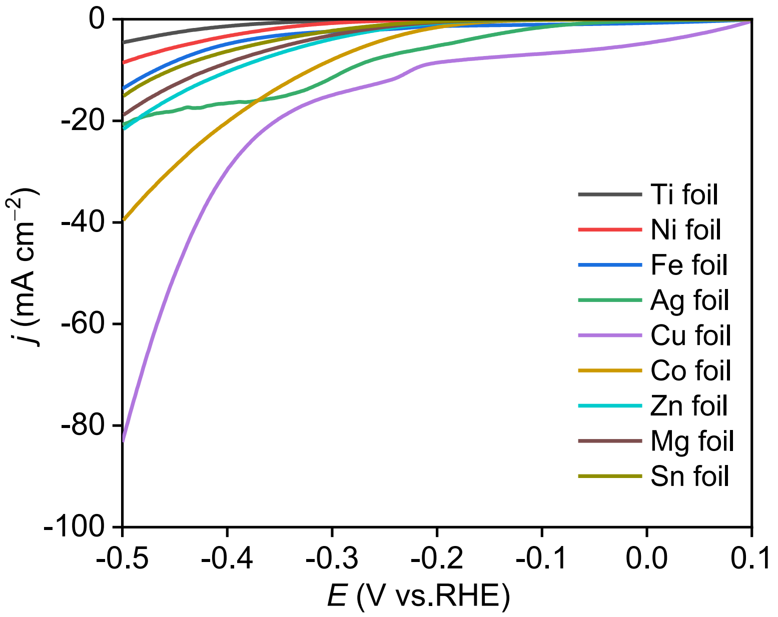

Fig. S1. LSV curves for the co-reduction of furfural and NO_3^-. Ti, Ni, Fe, Ag, Cu, Co, Zn, Mg, and Sn foil.

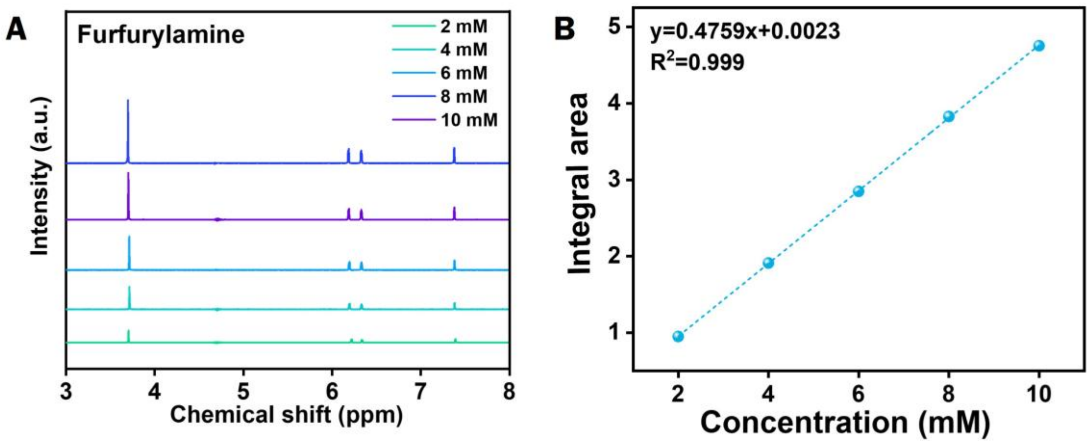

Fig. S2. Quantification for furfurylamine by ^{1}H NMR spectra. (A) ^{1}H NMR spectra of furfurylamine at various concentrations. **(B)** The calibration curve for its quantification.

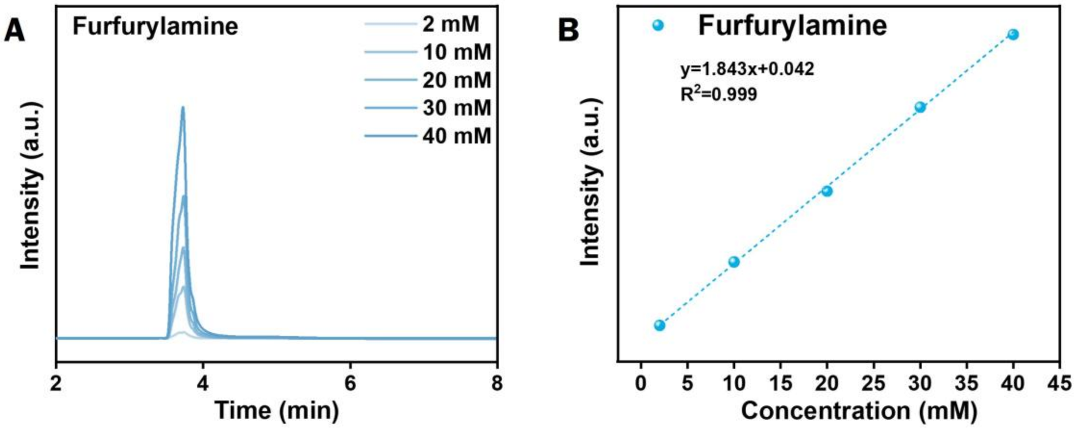

Fig. S3. Quantification for furfurylamine by HPLC spectra. (A) HPLC spectra of furfurylamine at various concentrations. **(B)** The calibration curve for its quantification.

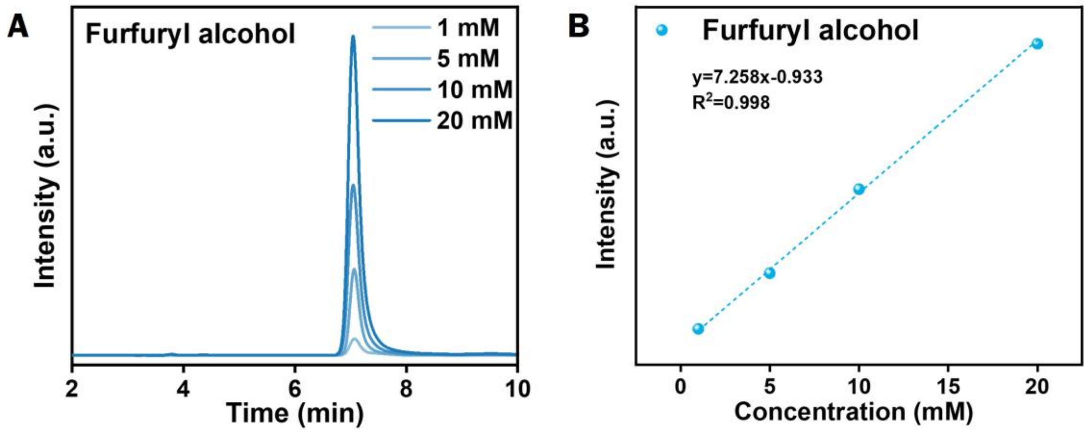

Fig. S4. Quantification for furfuryl alcohol by HPLC spectra. (A) HPLC spectra of furfuryl alcohol at various concentrations. **(B)** The calibration curve for its quantification.

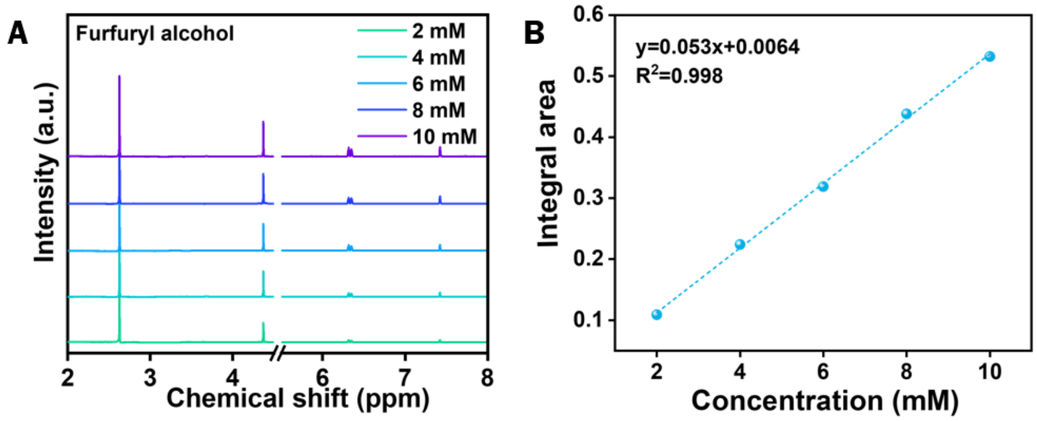

Fig. S5. Quantification for furfuryl alcohol by ^{1}H NMR spectra. (A) ^{1}H NMR spectra of furfuryl alcohol at various concentrations. **(B)** The calibration curve for its quantification.

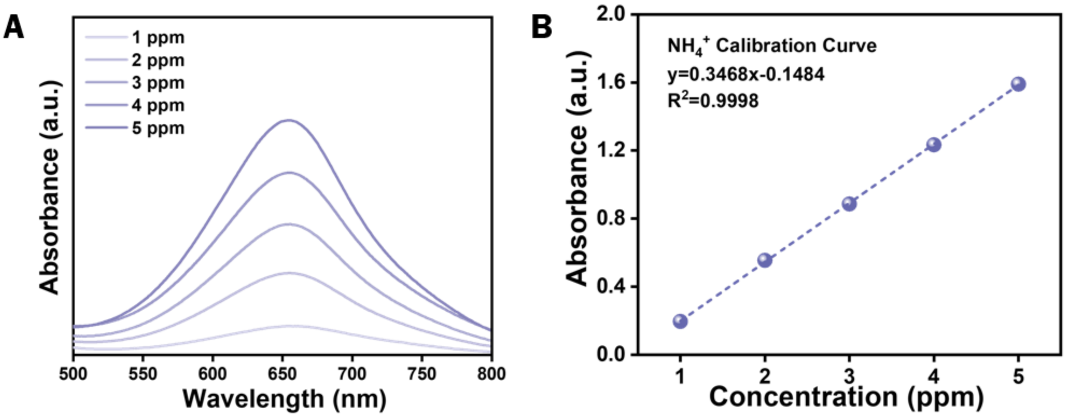

Fig. S6. Quantification for NH_4^+ by UV-vis absorption spectra. (A) UV-vis absorption spectra at various NH_4^+ concentrations. **(B)** The calibration curve for its quantification.

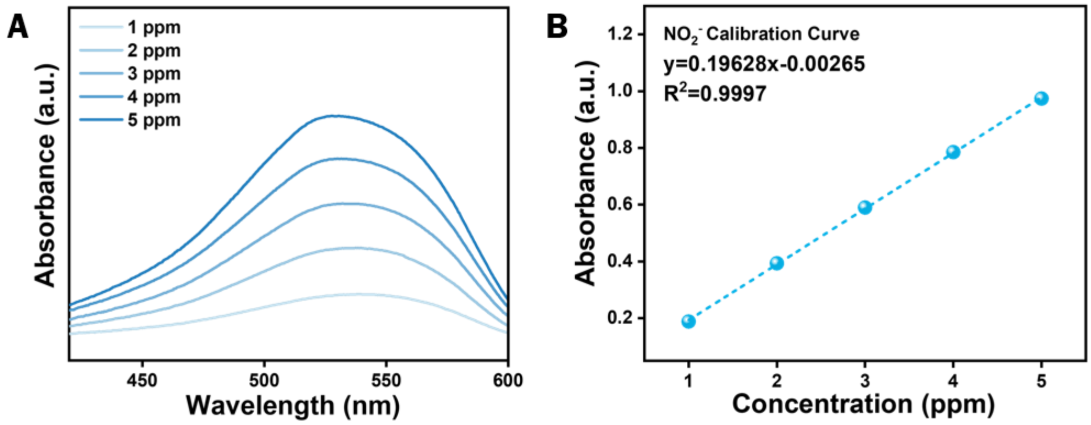

Fig. S7. Quantification for NO_2^- by UV-vis absorption spectra. **(A)** UV-vis absorption spectra at various NO_2^- concentrations. **(B)** The calibration curve for its quantification.

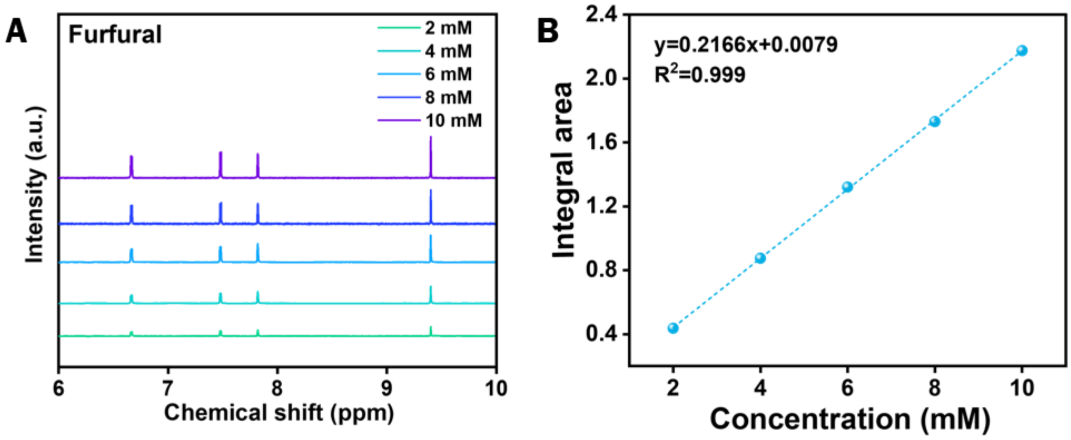

Fig. S8. Quantification for furfural by ^{1}H NMR spectra. (A) ^{1}H NMR spectra at various furfural concentrations. **(B)** The calibration curve for its quantification.

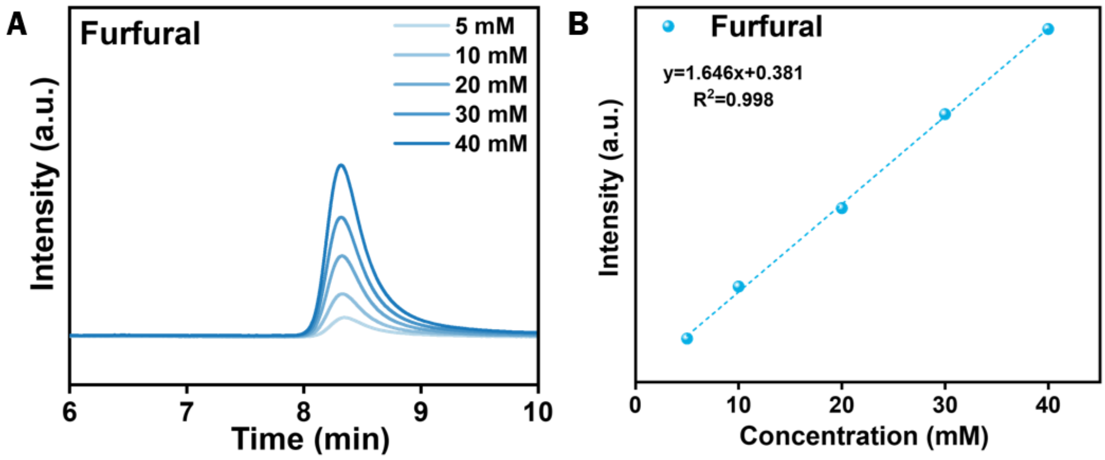

Fig. S9. Quantification for furfural by HPLC spectra. (A) HPLC at various furfural concentrations. **(B)** The calibration curve for its quantification.

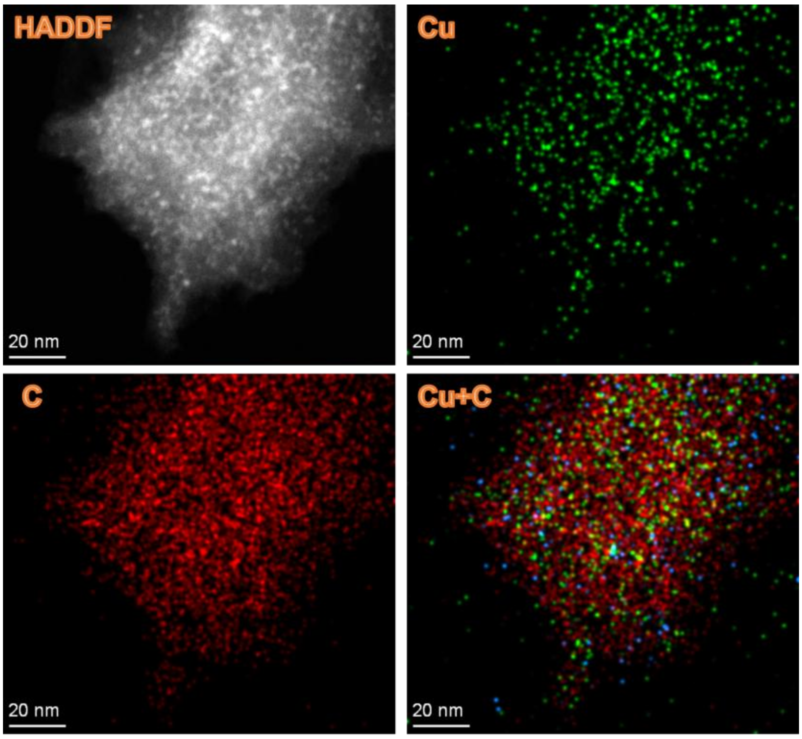

Fig. S10. HADDF-STEM images and corresponding elemental mapping of Cu-SNC.

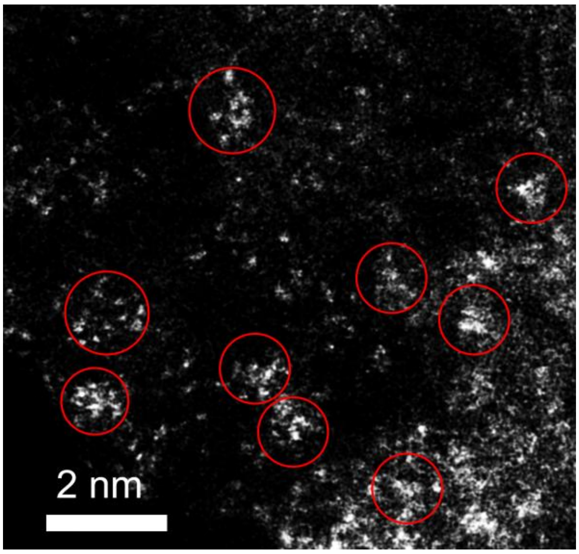

Fig. S11. High-resolution HADDF-STEM images Cu-SNC.

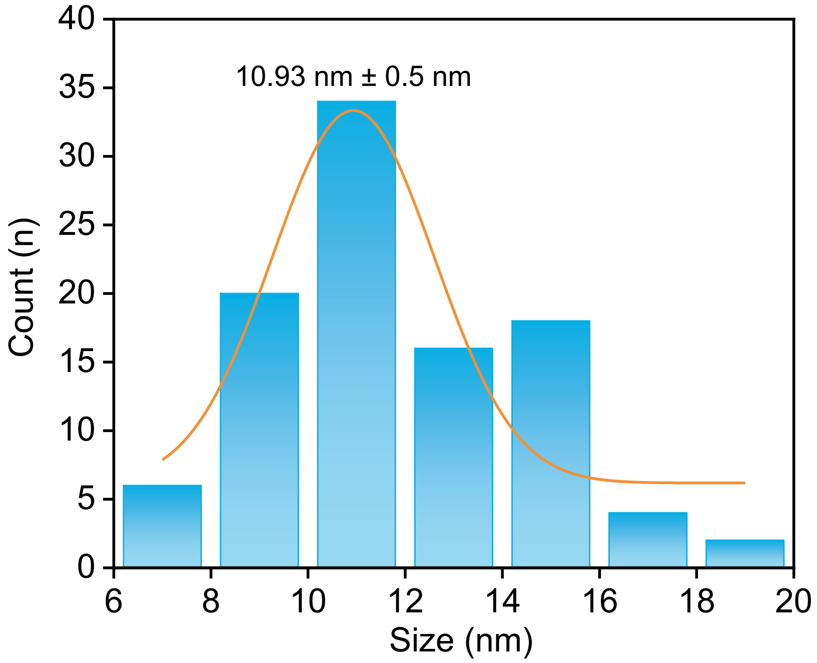

Fig. S12. Size distribution of Cu nanoparticles in Cu-NPs.

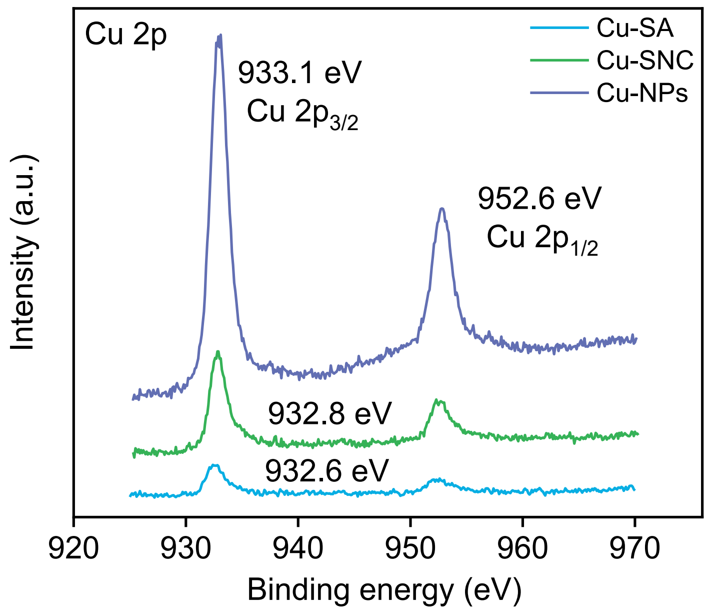

Fig. S13. XPS spectra of Cu 2p for Cu-SA, Cu-NSC, and Cu-NPs.

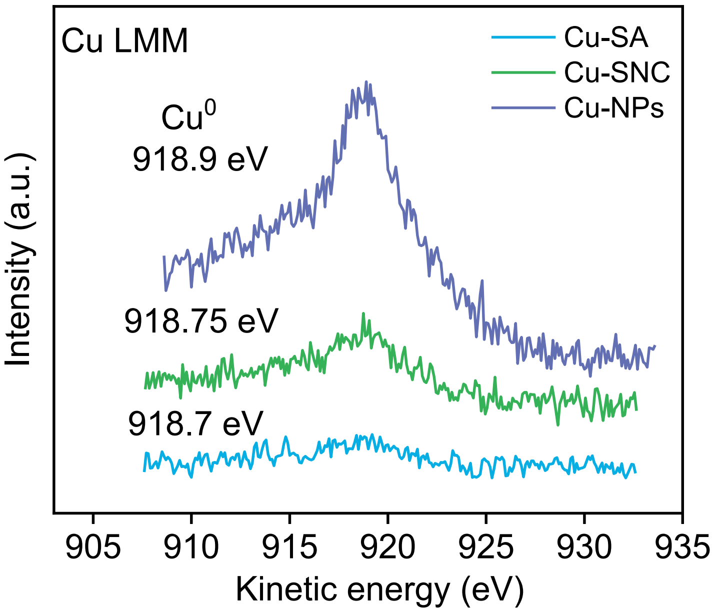

Fig. S14. XPS spectra of Cu LMM for Cu-SA, Cu-NSC, and Cu-NPs.

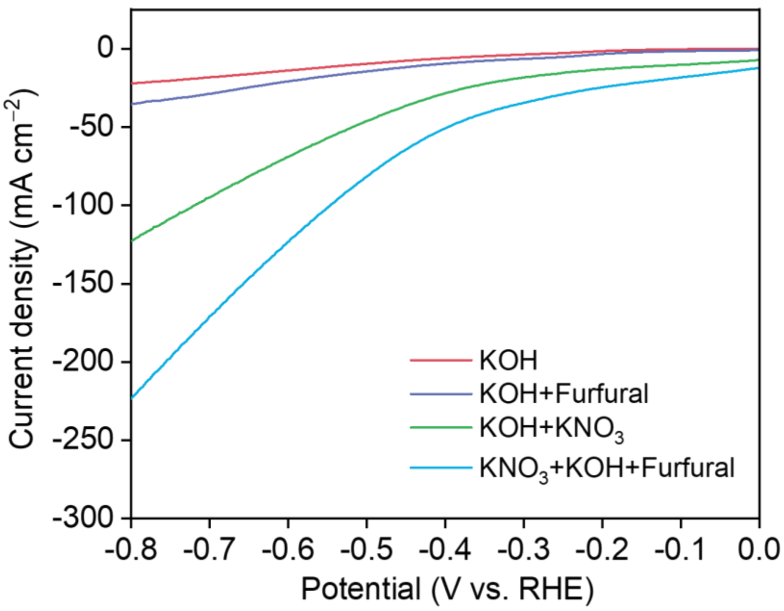

Fig. S15. LSV curves of Cu-SA in different electrolyte systems.

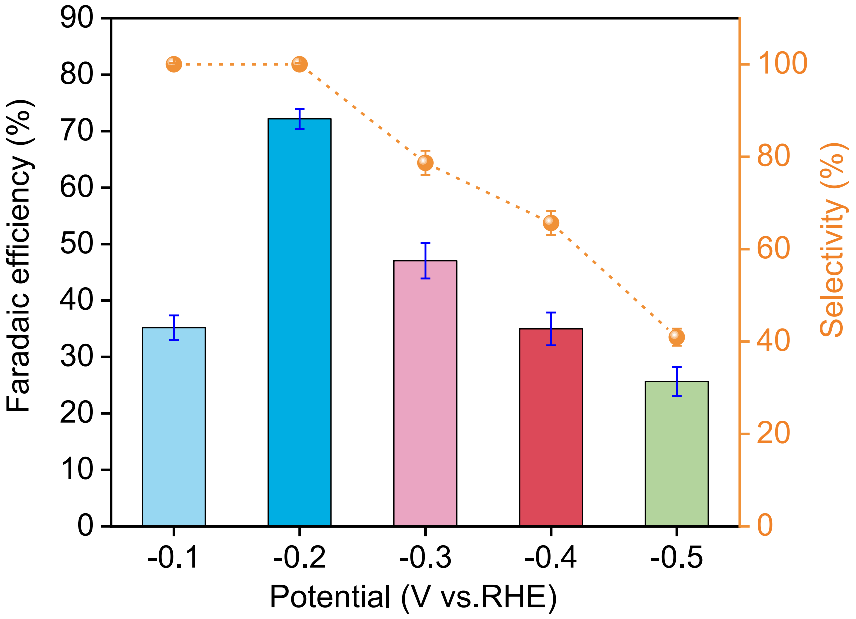

Fig. S16. Faradaic efficiency and selectivity of furfurylamine at different potentials.

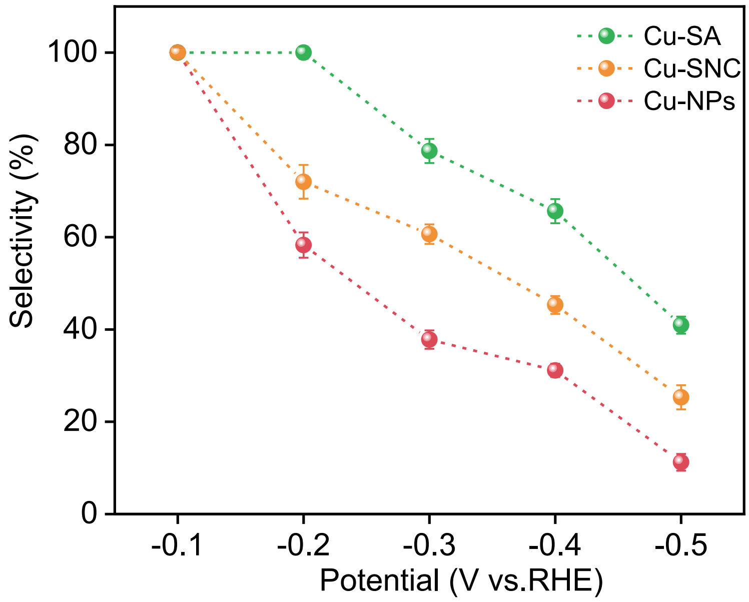

Fig. S17. Selectivity of furfurylamine over Cu-SA, Cu-SNC and Cu-NPs at different potentials.

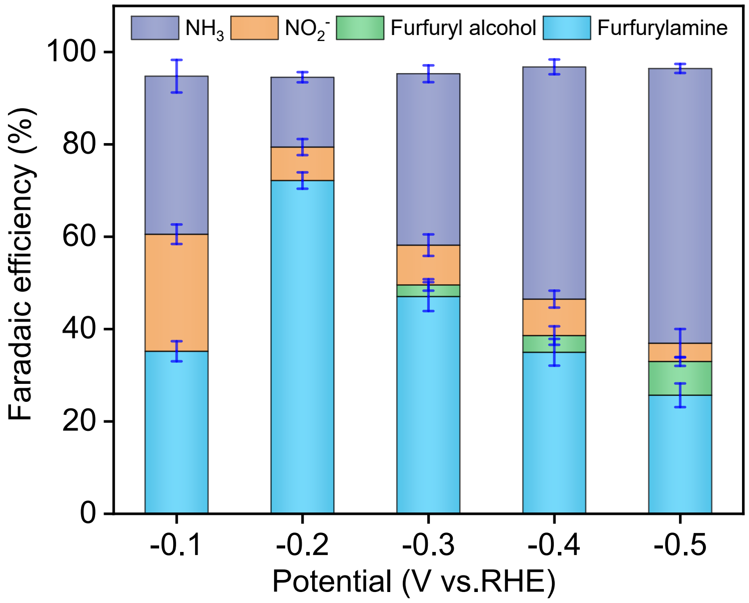

Fig. S18. Faradaic efficiencies of various products over Cu-SA at different potentials.

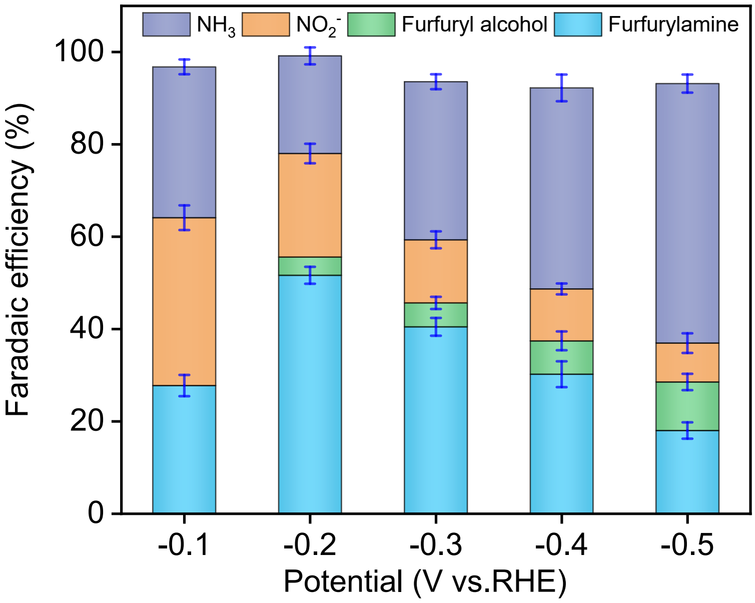

Fig. S19. Faradaic efficiencies of various products over Cu-SNC at different potentials.

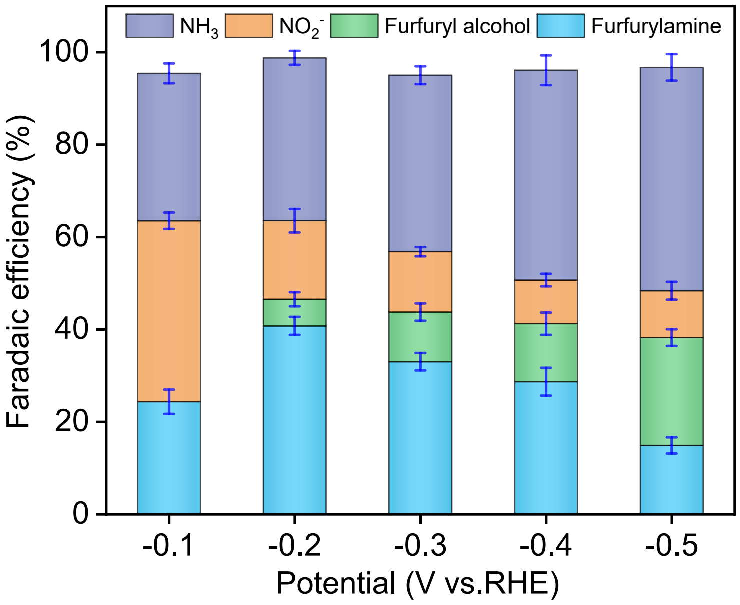

Fig. S20. Faradaic efficiencies of various products over Cu-NPs at different potentials.

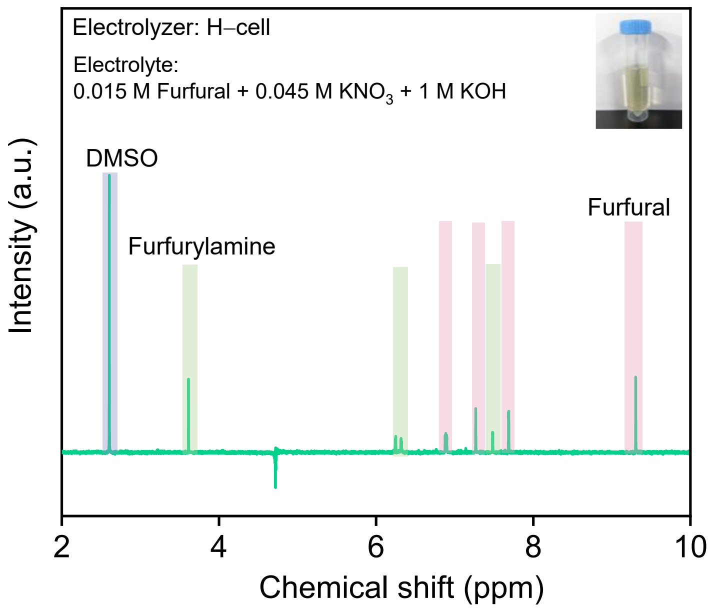

Fig. S21. ^{1}H NMR spectrum of the liquid product collected from H-cell tests. The electrolyte consisted of 0.015 M furfural, 0.045 M KNO_3 and 1 M KOH, with the reaction performed at −0.2 V vs. RHE; the inset shows a photograph of the liquid product.

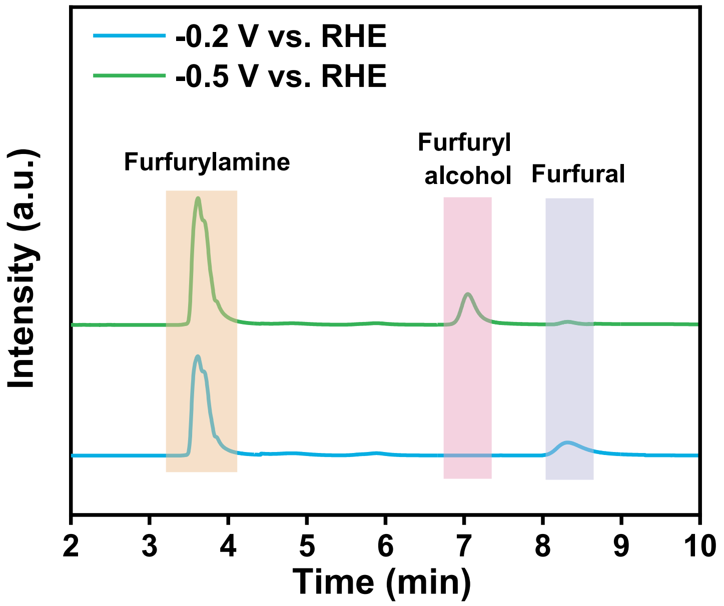

Fig. S22. **HPLC spectra of liquid products collected from the co-reduction at the potentials of –0.2 and –0.5 V vs. RHE.**

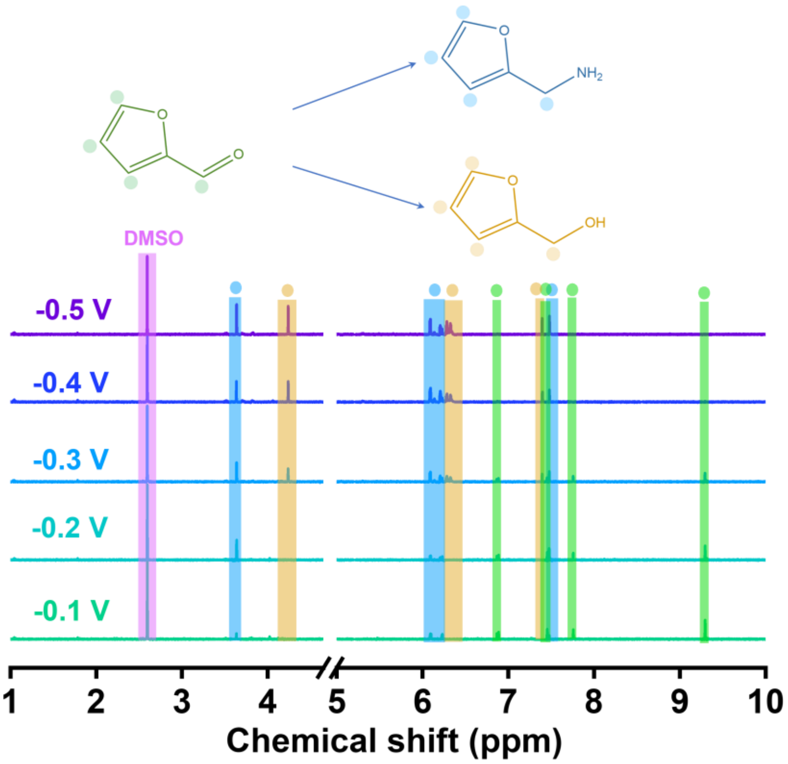

Fig. S23. **^{1}H NMR spectra of liquid products collected from the co-reduction at potentials ranging from –0.1 to –0.5 V vs. RHE.**

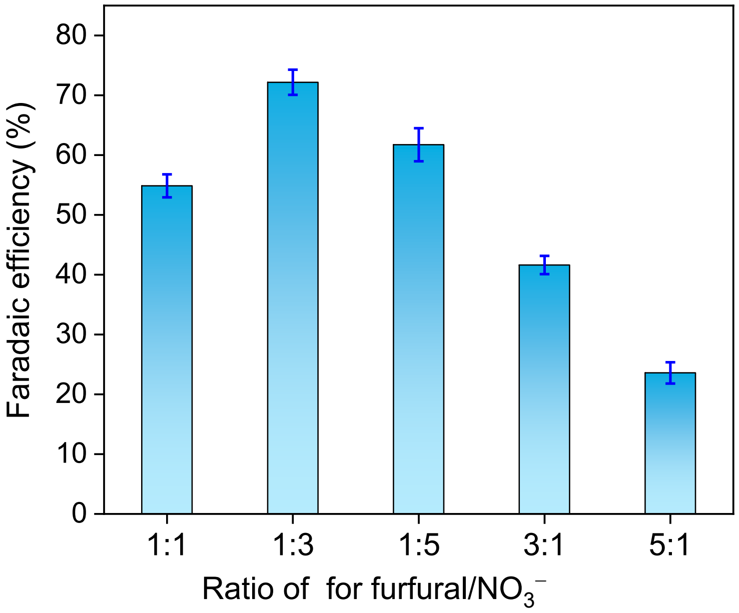

Fig. S24. Faradaic efficiencies of furfurylamine for the co-reduction of furfural and NO_3^- with different molar ratio at −0.2 V vs. RHE

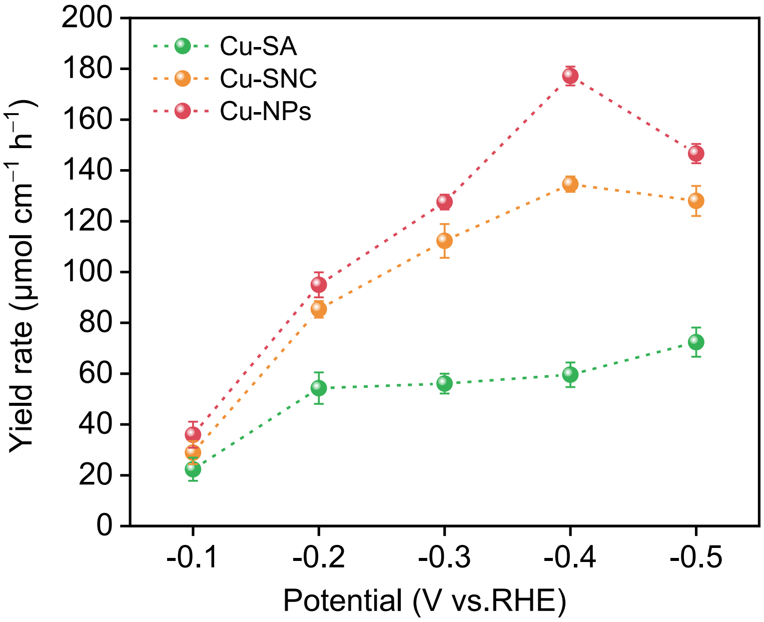

Fig. S25. Yield rates of furfurylamine for the co-reduction of furfural and NO_3^- over Cu-SA, Cu-SNC, and Cu-NPs at different potential.

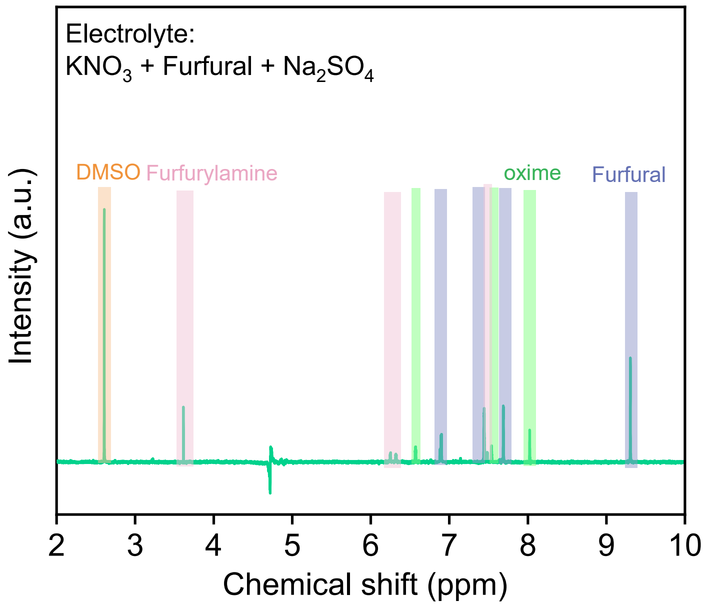

Fig. S26. **^{1}H NMR spectra of the liquid product collected at −0.2 V vs. RHE over Cu-SA, where a neutral electrolyte was used (i.e., 0.015 furfural, 0.045 M KNO_3, and 0.5 M Na_2SO_4).**

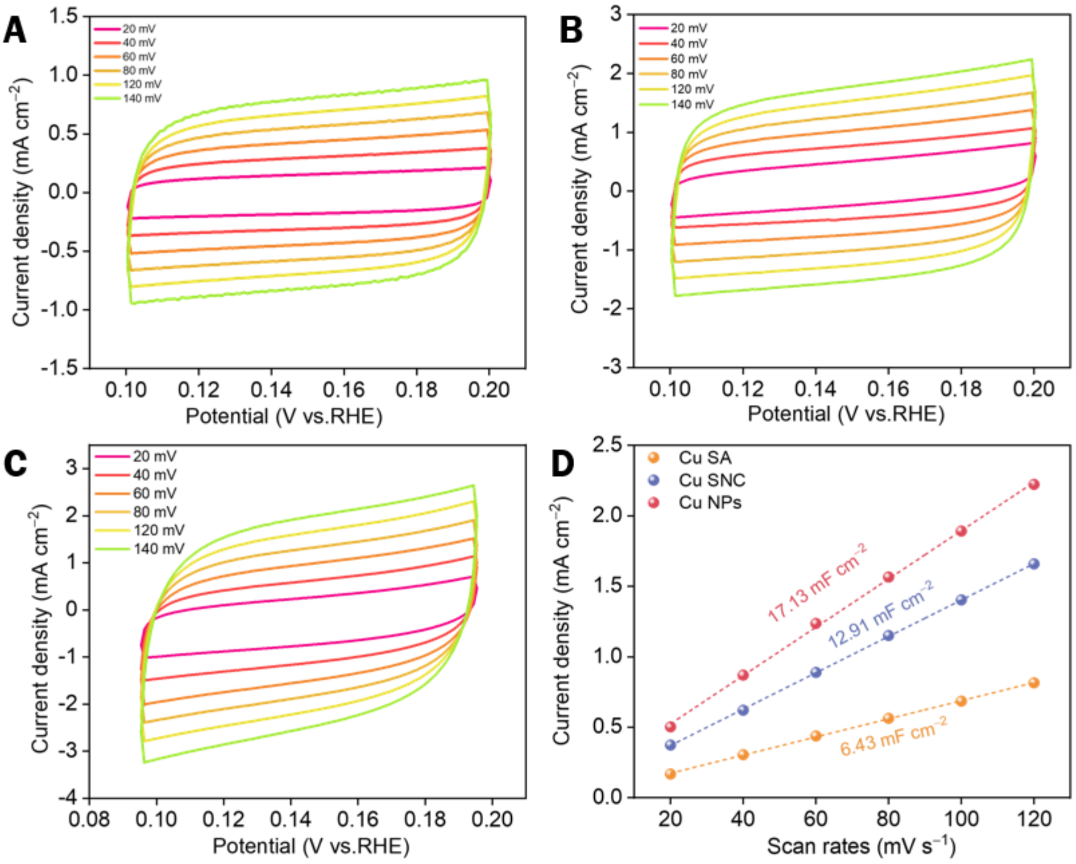

Fig. S27. CV curves of various catalysts at various scan rates within the non-faradic potential range. (A) Cu-SA, **(B)** Cu-SNC and **(C)** Cu-NPs **(D)** Plots of the current density versus the scan rate for the above noted three catalysts.

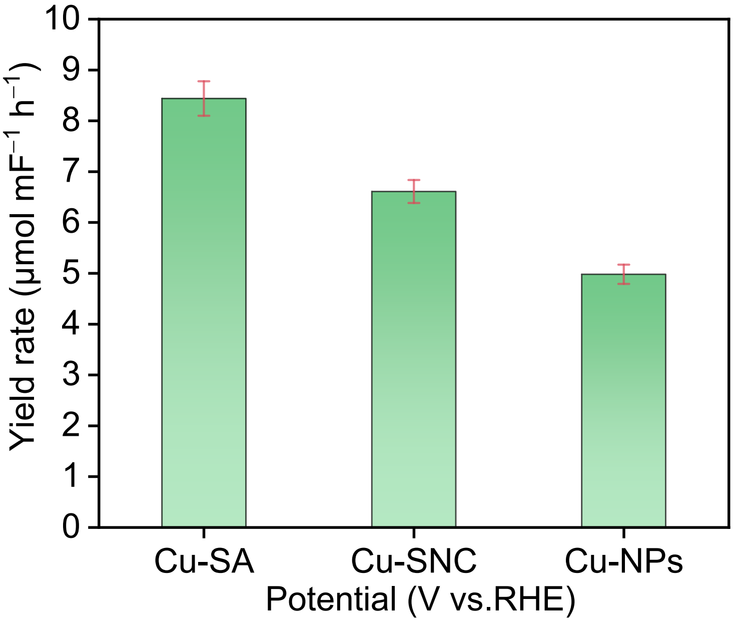

Fig. S28. The yield rate of furfurylamine by normalizing C_{dl} values over Cu-SA, Cu-SNC, and Cu-NPs at −0.2 V vs. RHE.

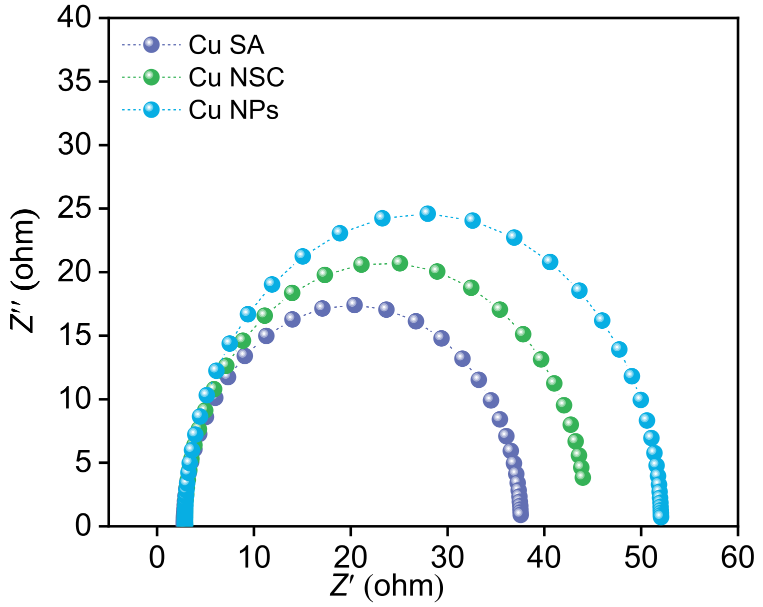

Fig. S29. EIS of Cu-SA, Cu-SNC and Cu-NPs at the potential of −0.2 V vs. RHE.

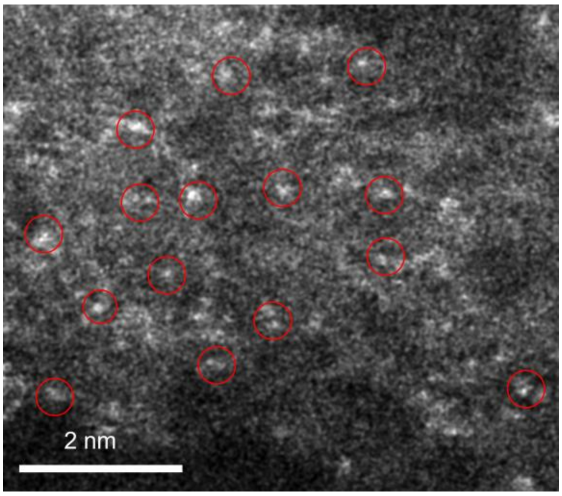

Fig. S30. HADDF-STEM images of Cu-SA after 20 cycles electrolysis.

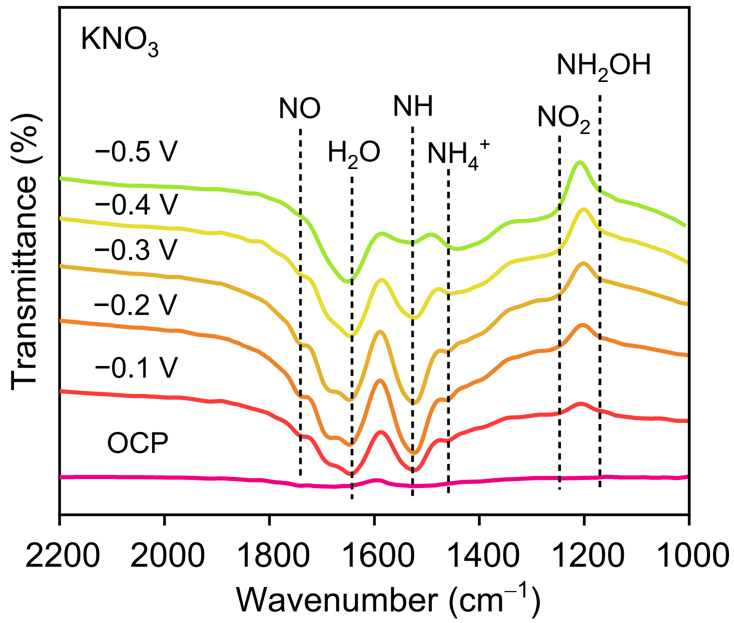

Fig. S31. Operando ATR-FTIR spectroscopy measurements for the individual NO_3^-RR under various potentials.

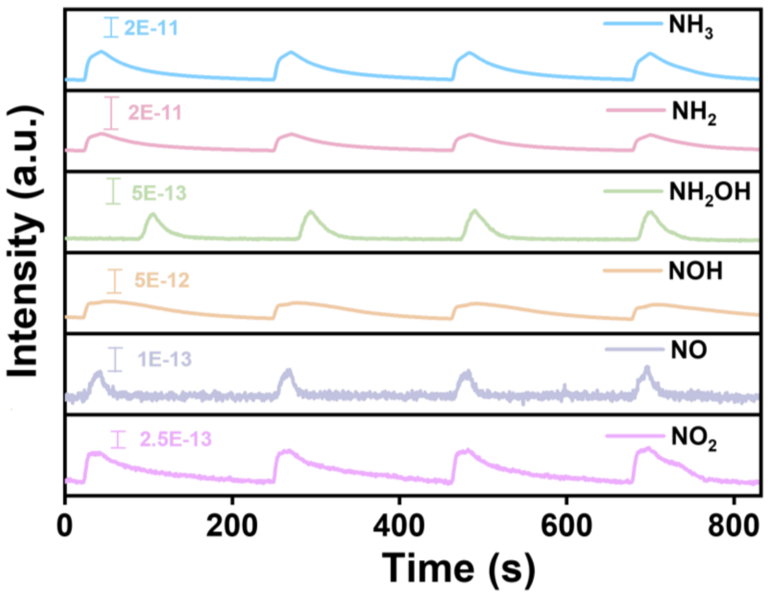

Fig. S32. Operando DEMS signals of Cu-SA for the individual NO_3^-RR at −0.2 V vs. RHE.

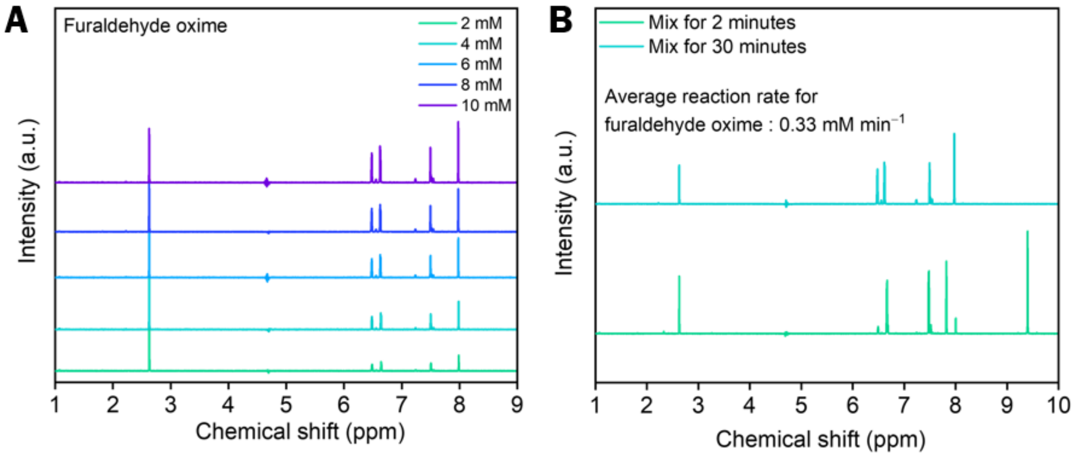

Fig. S33. Quantification for furaldehyde oxime by ^{1}H NMR spectra. (A) ^{1}H NMR spectra of furaldehyde oxime at various concentrations. **(B)** ^{1}H NMR spectra of the hydroxylamine/furfural mixture recorded at 2 minutes and 30 minutes under ambient temperature and without an applied potential.

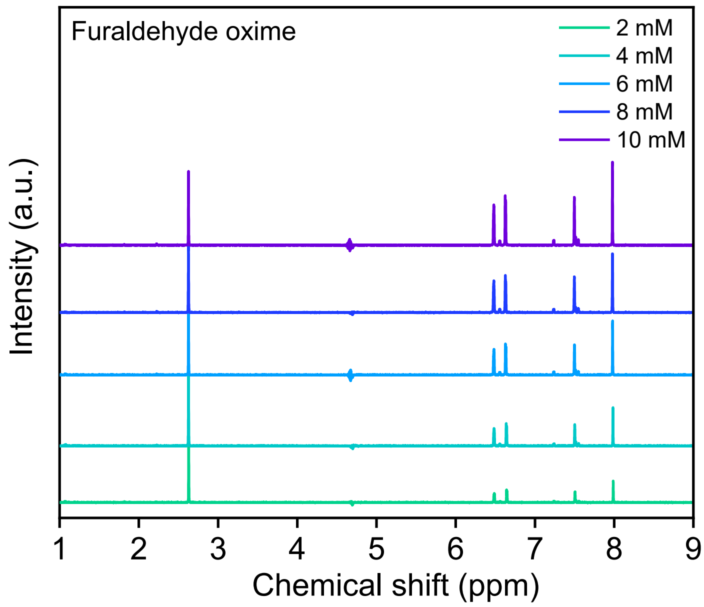

Fig. S34. ^{1}**H NMR spectra of furaldehyde oxime at various concentration.**

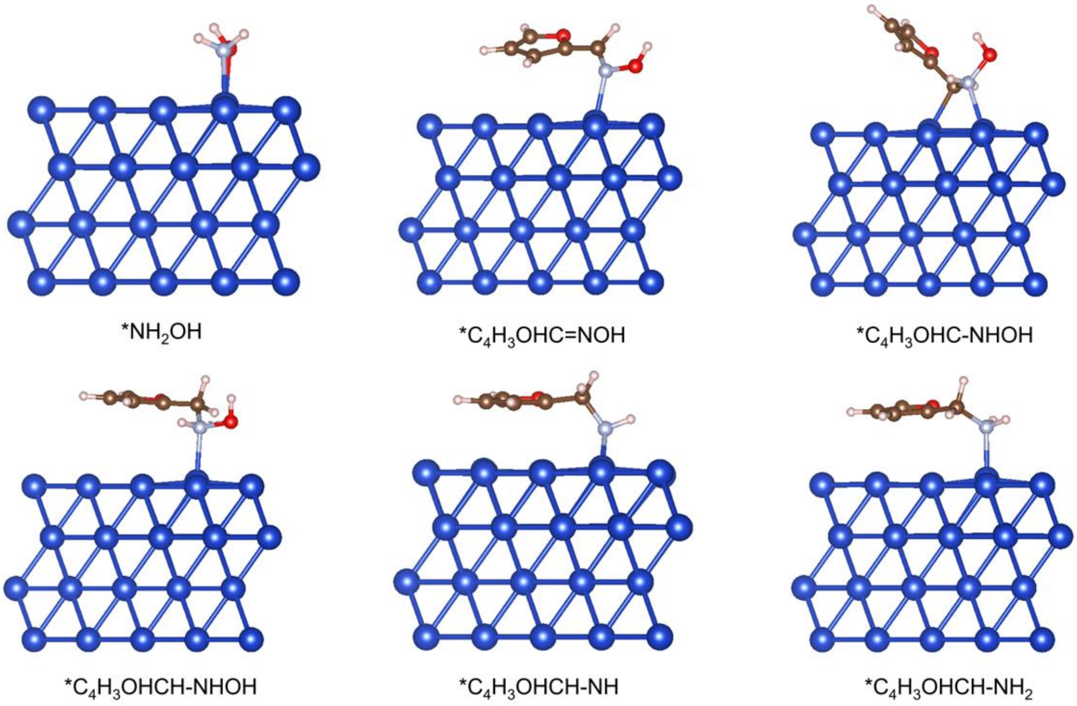

Fig. S35. The atomic models for Cu-NPs. Side view of all the intermediates adsorption on the surface of Cu-NPs for the conversion of *NH_2OH to $C_4H_3OCH_2NH_2$.

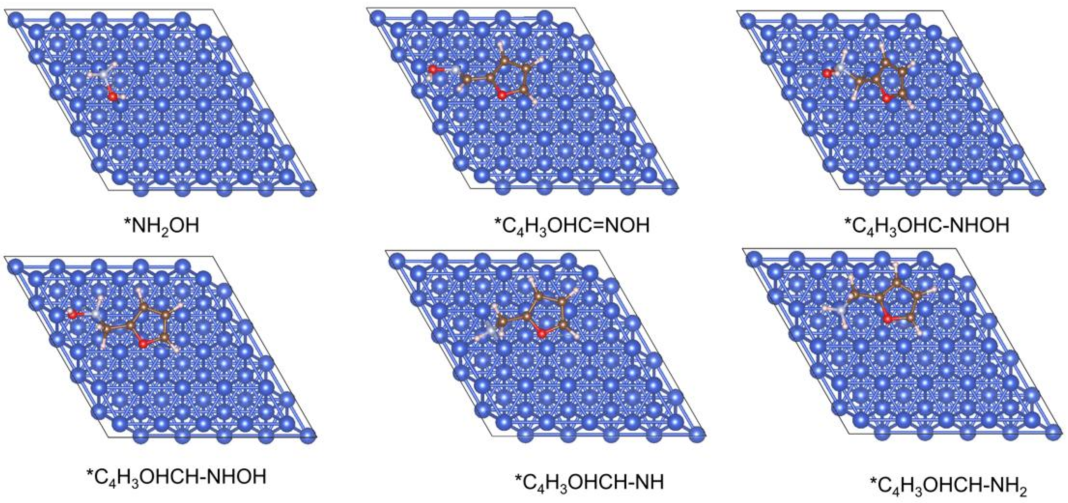

Fig. S36. The atomic models for Cu-NPs. Top view of all the intermediates adsorption on the surface of Cu-NPs for the conversion of *NH_2OH to $C_4H_3OCH_2NH_2$.

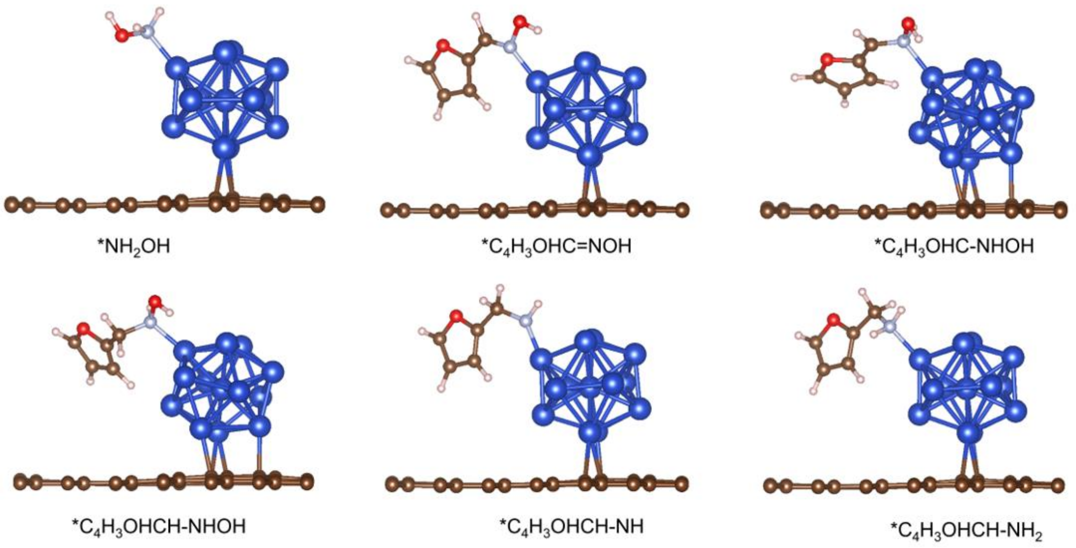

Fig. S37. The atomic models for Cu-SNC. Side view of all the intermediates adsorption on the surface of Cu-SNC for the conversion of *NH_2OH to $C_4H_3OCH_2NH_2$.

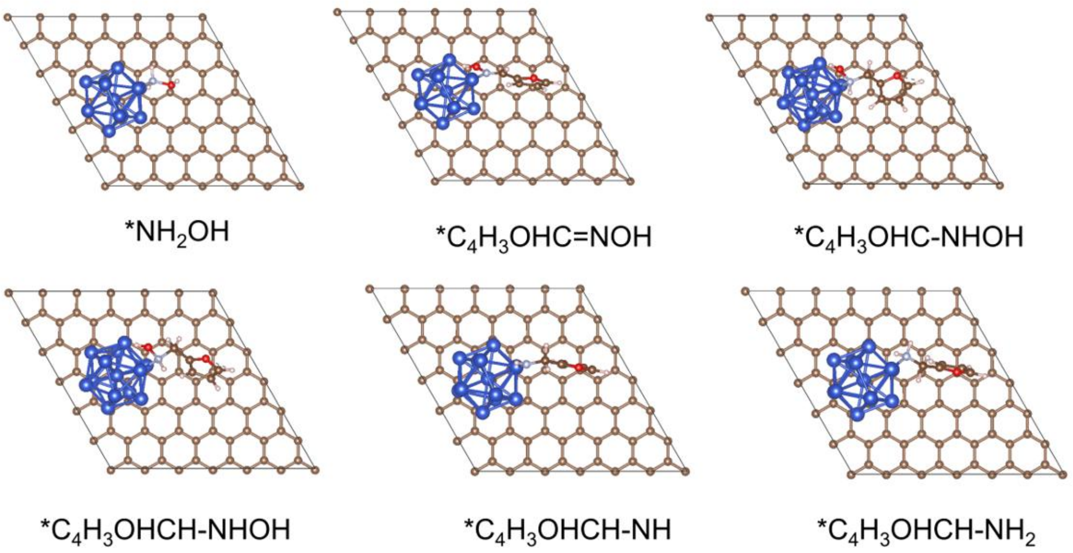

Fig. S38. The atomic models for Cu-SNC. Top view of all the intermediates adsorption on the surface of Cu-SNC for the conversion of $*NH_2OH$ to $C_4H_3OCH_2NH_2$.

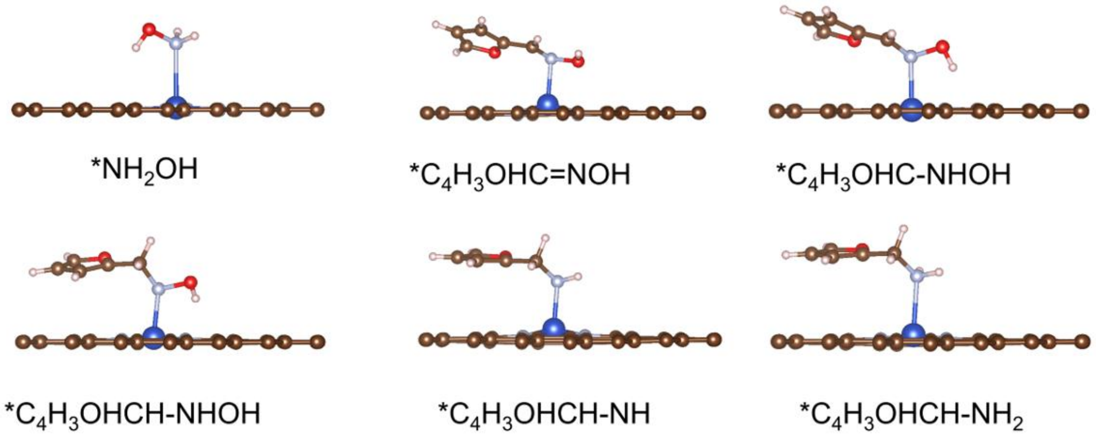

Fig. S39. The atomic models for Cu-SA. Side view of all the intermediates adsorption on the surface of Cu-SA for the conversion of $*NH_2OH$ to $C_4H_3OCH_2NH_2$.

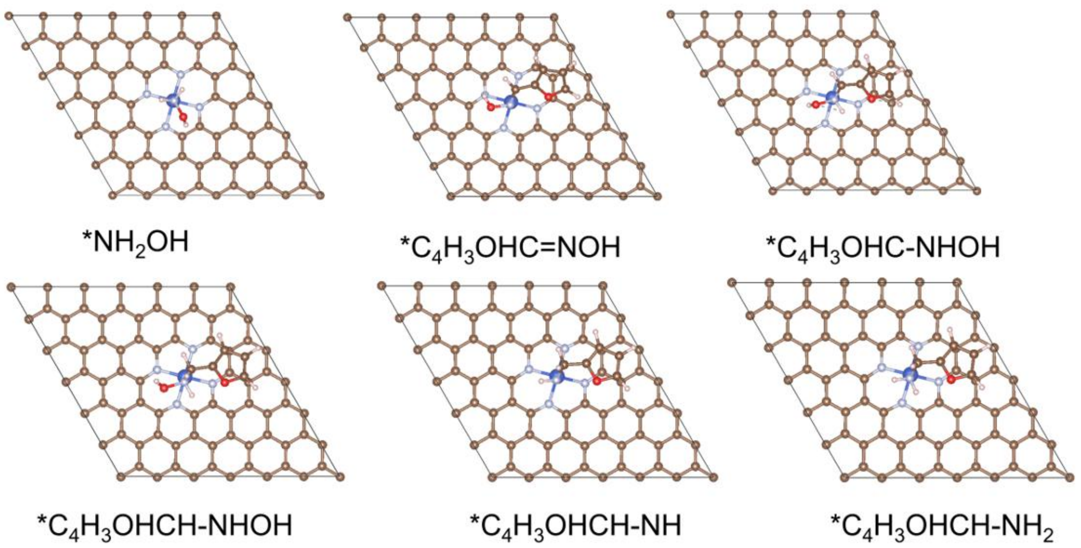

Fig. S40. The atomic models for Cu-SA. Top view of all the intermediates adsorption on the surface of Cu-SA for the conversion of *NH_2OH to $C_4H_3OCH_2NH_2$.

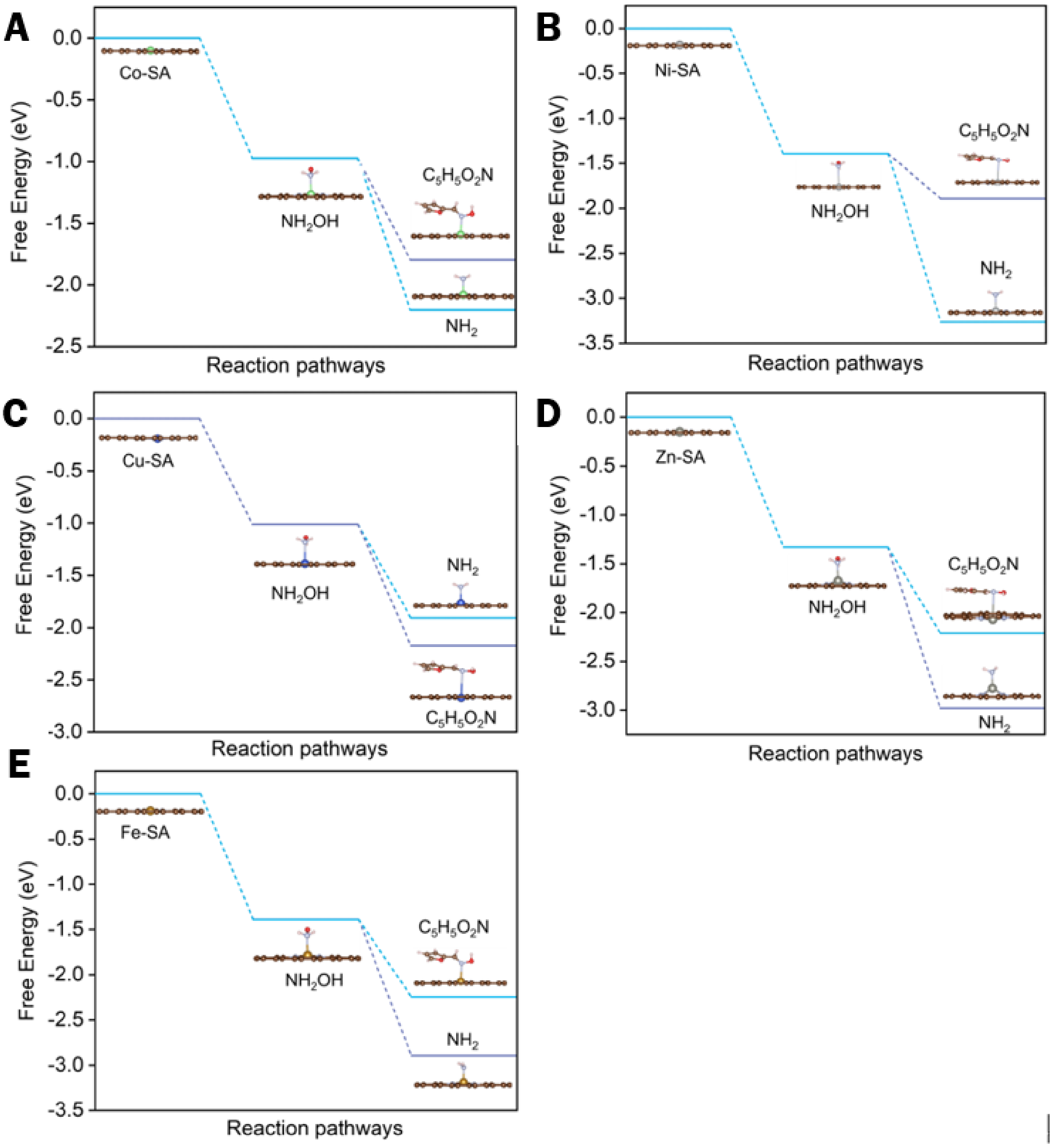

Fig. S41. Thermodynamic energy barriers for the conversion of hydroxylamine to NH_2 and the C-N coupling between hydroxylamine and furfural. (A) Co-SA. **(B)** Ni-SA. **(C)** Cu -SA. **(D)** Zn-SA. **(E)** Fe-SA.

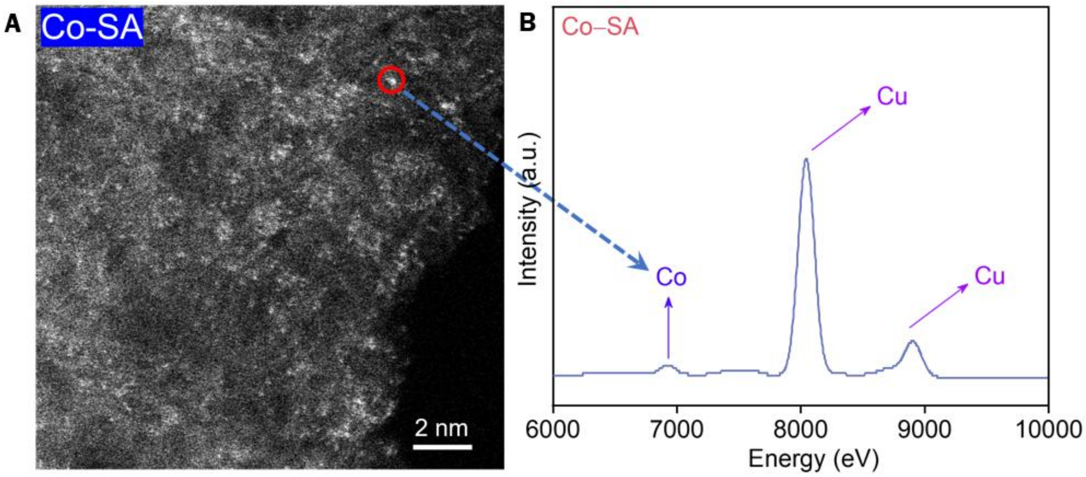

Fig. S42. Morphological characterization of Co-SA. (A) HADDF-STEM images of Co-SA and **(B)** corresponding EDX point scanning image (The peak of Co can be assigned to Co -SA and the peak of Cu can be assigned to be the background of Cu micro grid).

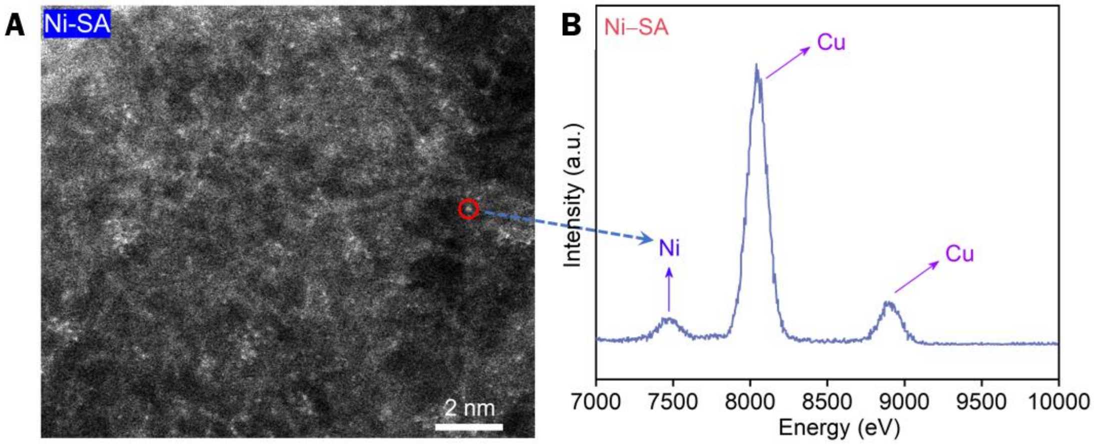

Fig. S43. Morphological characterization of Ni-SA. (A) HADDF-STEM images of Ni-SA and **(B)** corresponding EDX point scanning image (The peak of Ni can be assigned to Ni-SA and the peak of Cu can be assigned to be the background of Cu micro grid).

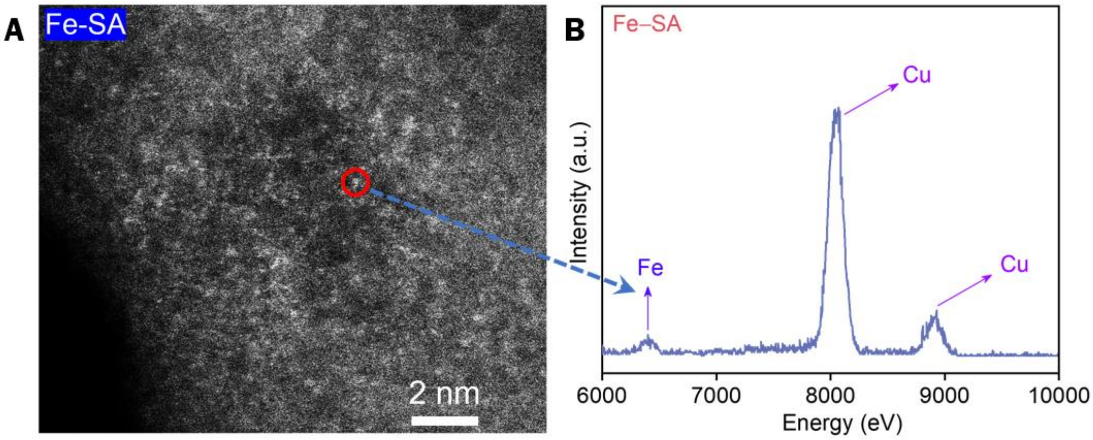

Fig. S44. Morphological characterization of Fe-SA (A) HADDF-STEM images of Fe-SA and **(B)** corresponding EDX point scanning image (The peak of Fe can be assigned to Fe-SA and the peak of Cu can be assigned to be the background of Cu micro grid).

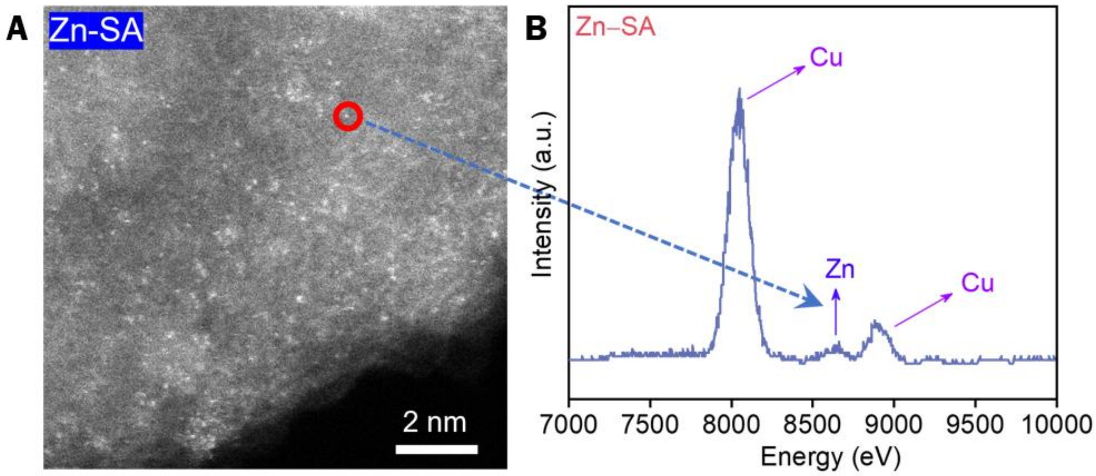

Fig. S45. Morphological characterization of Zn-SA (A) HADDF-STEM images of Zn-SA and **(B)** corresponding EDX point scanning image (The peak of Zn can be assigned to Zn -SA and the peak of Cu can be assigned to be the background of Cu micro grid).

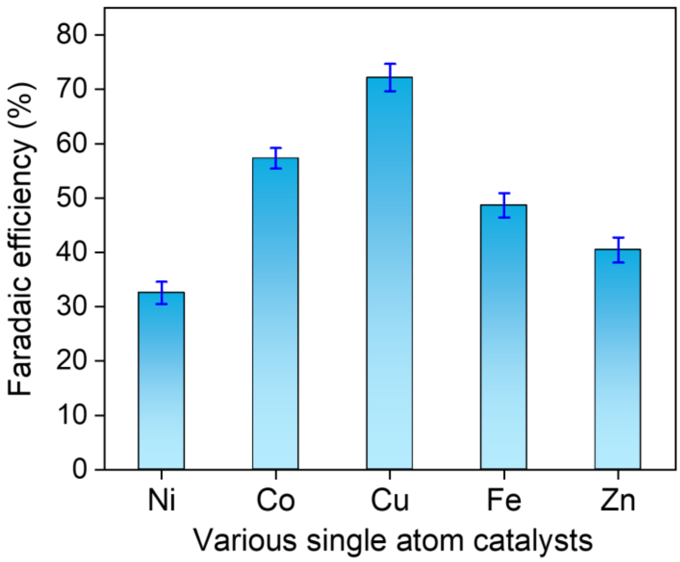

Fig. S46. Faradaic efficiency for the co-reduction of NO_3^-/furfural over various single atomic metal catalysts at −0.2 V vs. RHE.

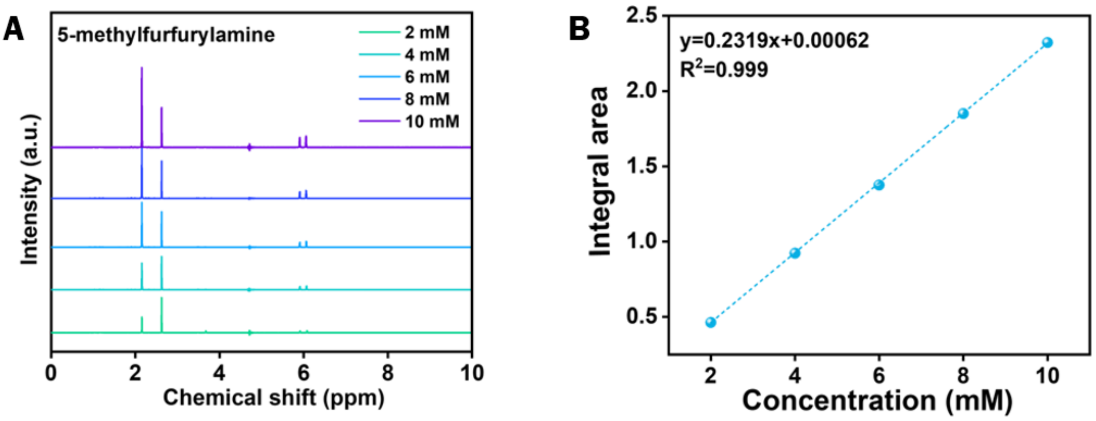

Fig. S47. Quantification for 5-mentheylfurfurylamine by ^{1}H NMR spectra. (A) ^{1}H NMR spectra of spectra of 5-mentheylfurfurylamine at various concentrations. **(B)** The calibration curve for its quantification.

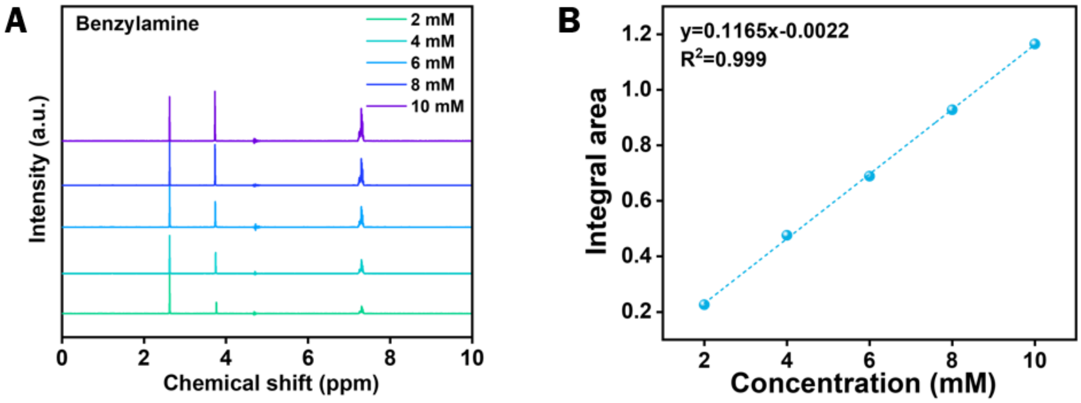

Fig. S48. Quantification for benzylamine by ^{1}H NMR spectra. (A) ^{1}H NMR spectra of spectra of benzylamine at various concentrations. **(B)** The calibration curve for its quantification.

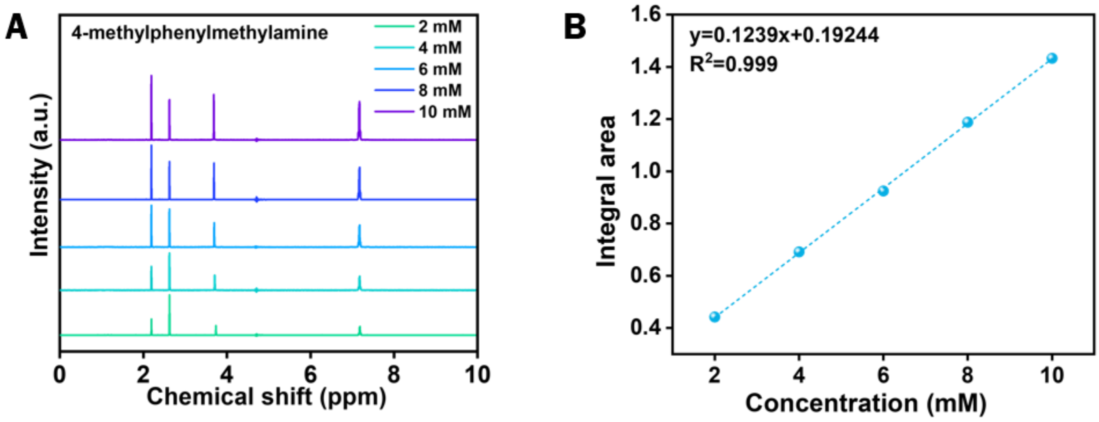

Fig. S49. Quantification for 4-methylphenylmethylamine by ^{1}H NMR spectra. (A) ^{1}H NMR spectra of spectra of 4-methylphenylmethylamine at various concentrations. **(B)** The calibration curve for its quantification.

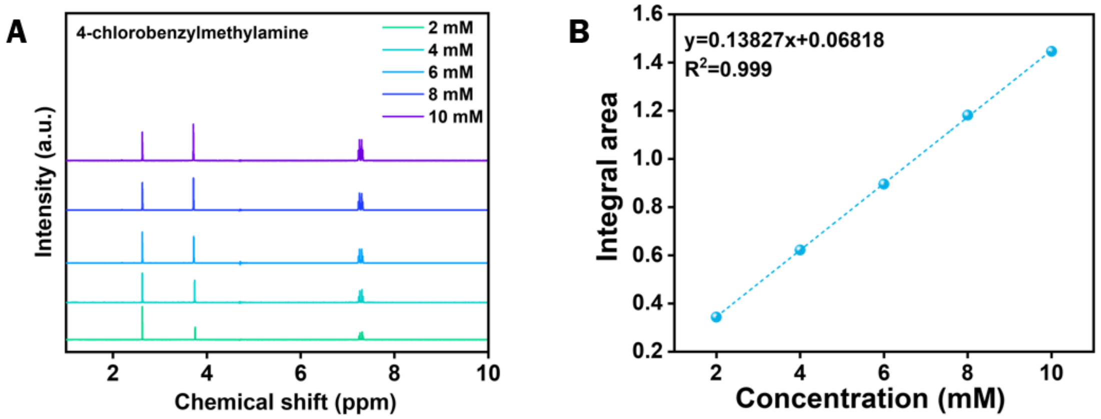

Fig. S50. Quantification for 4-chlorobenzylmethylamine by ^{1}H NMR spectra. (A) ^{1}H NMR spectra of spectra of 4-chlorobenzylmethylamine at various concentrations. **(B)** The calibration curve for its quantification.

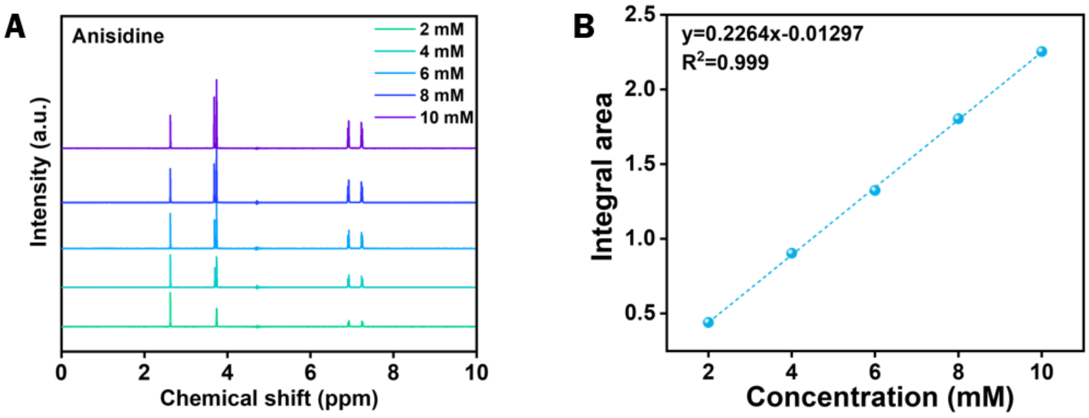

Fig. S51. Quantification for anisidine by ^{1}H NMR spectra. (A) ^{1}H NMR spectra of spectra of anisidine at various concentrations. **(B)** The calibration curve for its quantification

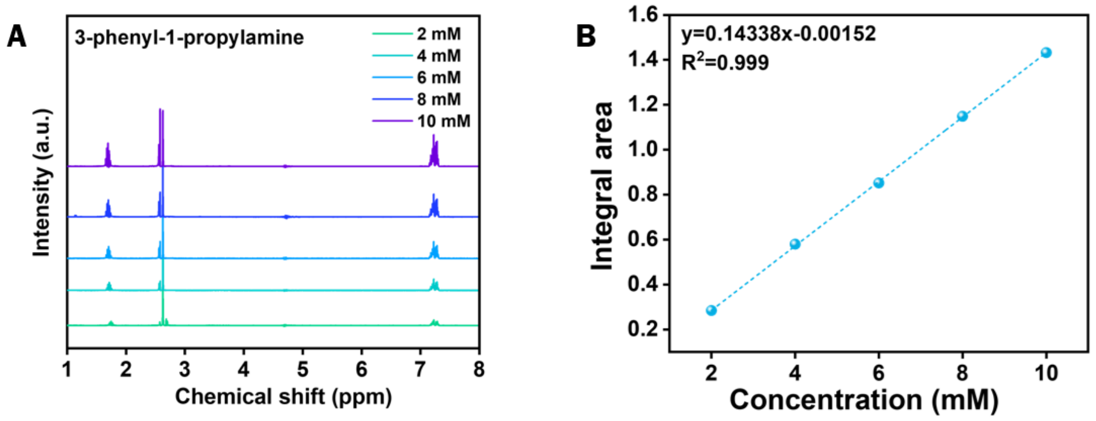

Fig. S52. Quantification for 3-phenyl-1-propylamine by 1H NMR spectra. (A) ^{1}H NMR spectra of spectra of 3-phenyl-1-propylamine at various concentrations. **(B)** The calibration curve for its quantification

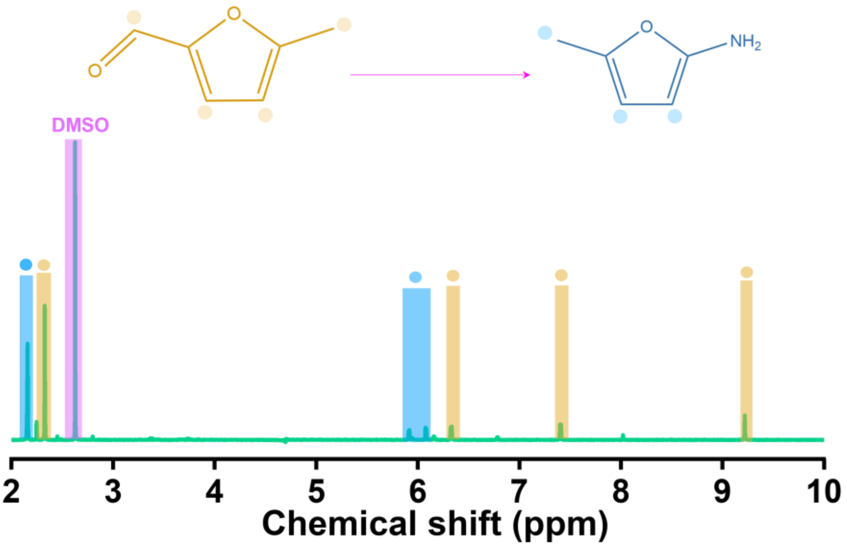

Fig. S53. ^{1}H NMR spectra of electrolytes after the co-reduction of 5-methylfurfural/NO_3^- at −0.2 V vs. RHE.

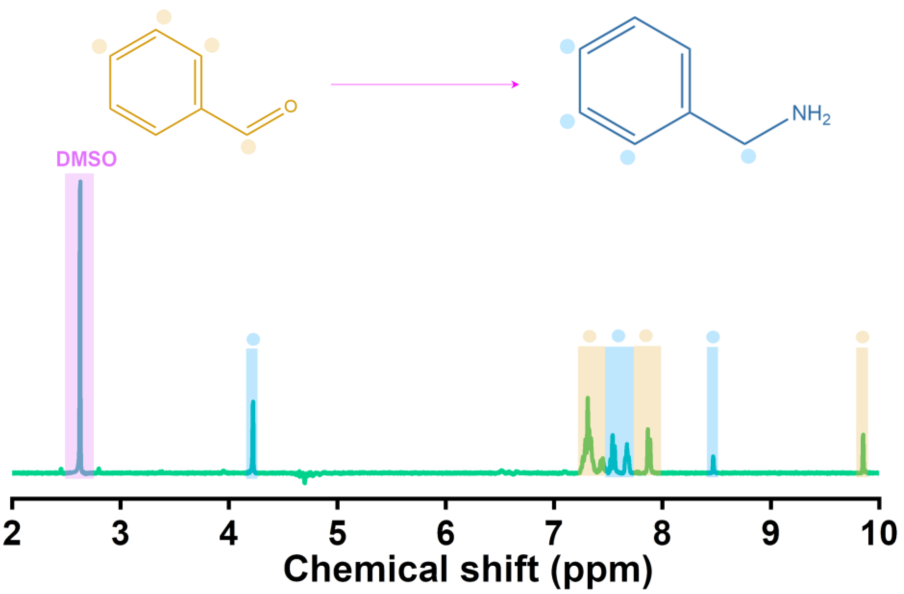

Fig. S54. ^{1}H NMR spectra of electrolytes after the co-reduction of benzaldehyde /NO_3^- at −0.2 V vs. RHE.

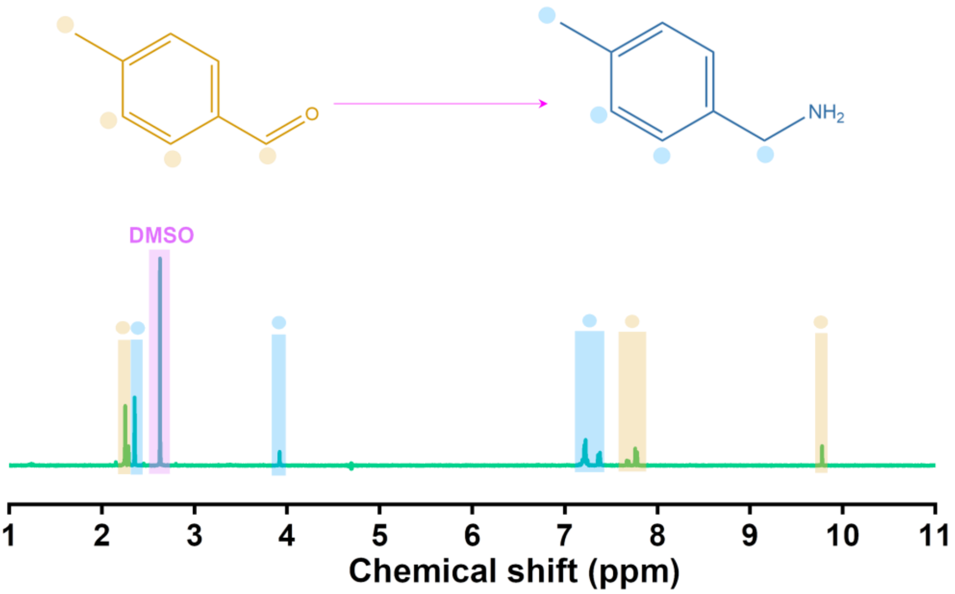

Fig. S55. [1]**H NMR spectra of electrolytes after the co-reduction of p-Methyl benzaldehyde /NO_3^- at −0.2 V vs. RHE.**

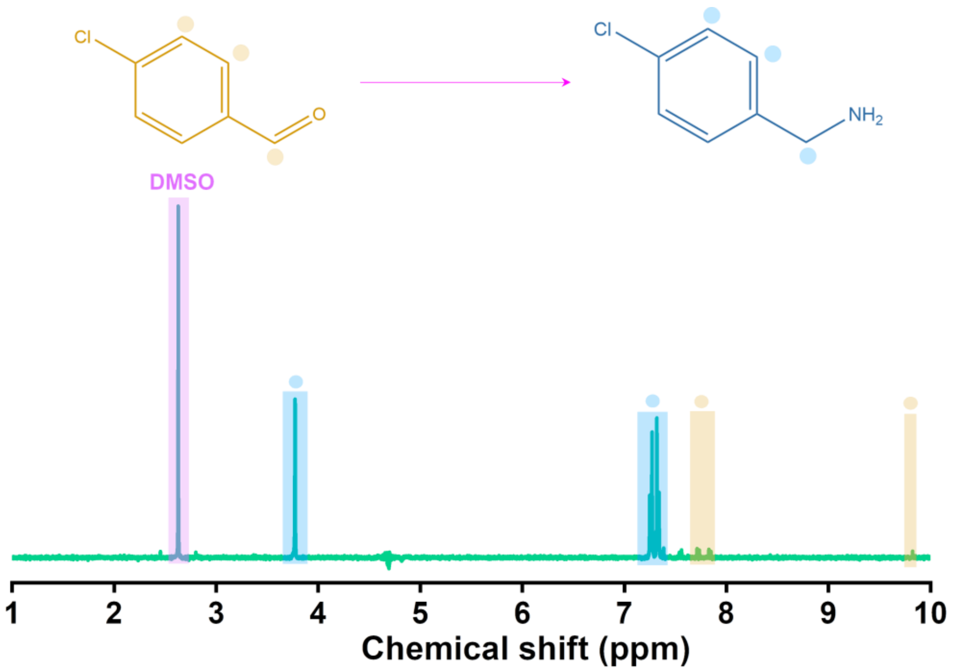

Fig. S56. ^{1}H NMR spectra of electrolytes after the co-reduction of 4-chlorobenzaldehyde /NO_3^- at −0.2 V vs. RHE.

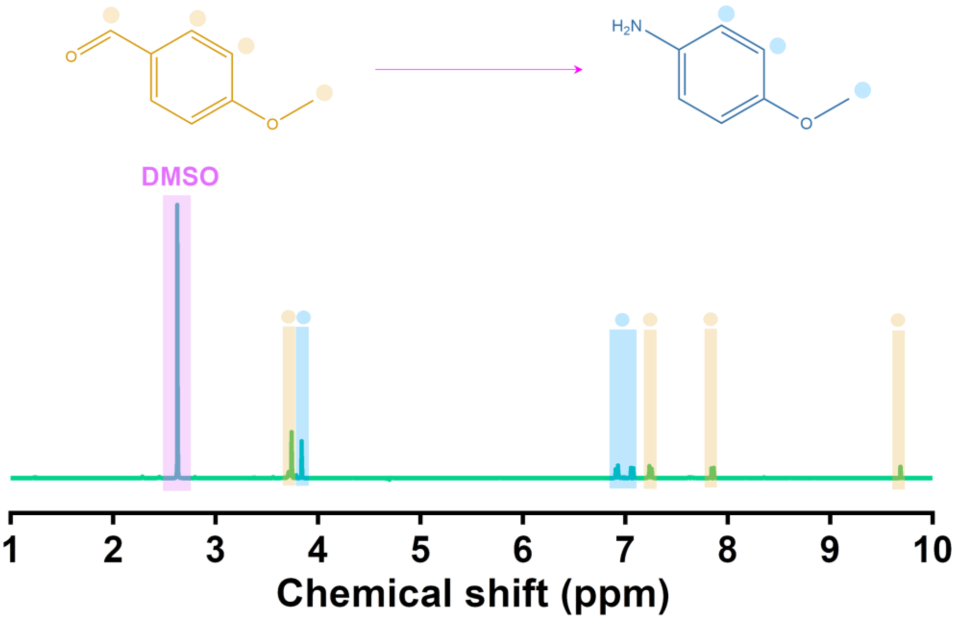

Fig. S57. **^{1}H NMR spectra of electrolytes after the co-reduction of anisaldehyde /NO_3^- at −0.2 V vs. RHE.**

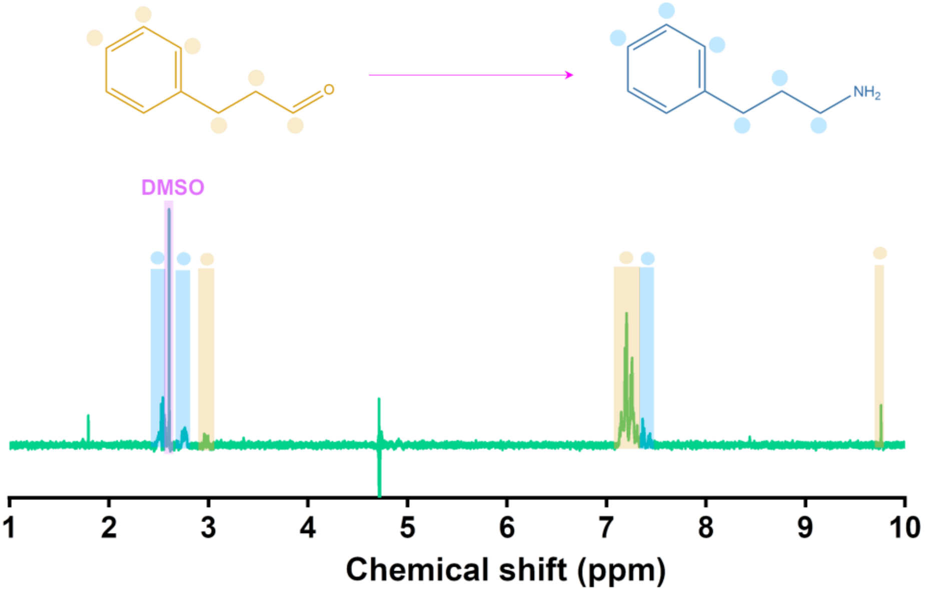

Fig. S58. ^{1}H **NMR spectra of electrolytes after the co-reduction of 3-phenyl-1-propanal/NO_3^- at −0.2 V vs. RHE.**

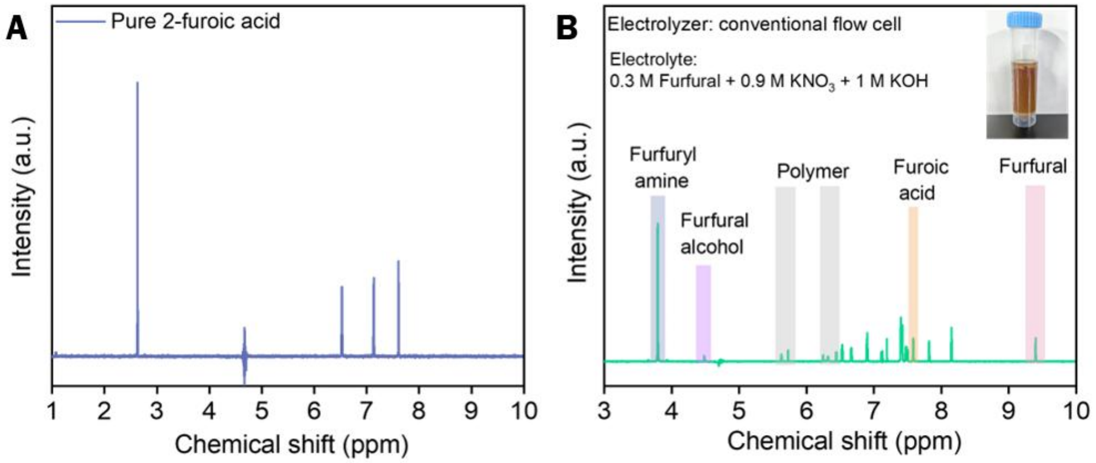

Fig. S59. ¹H NMR spectrum for 2-furoic acid and the liquid product. (A) ¹H NMR spectrum of pure 2-furoic acid. **(B)** ¹H NMR spectrum of the liquid product collected from conventional flow cell, where the electrolyte consisted of 0.3 M Furfural, 0.9 M KNO_3 and 1 M KOH, with the reaction performed at a cell voltage of 1.9 V; the inset shows a photograph of the liquid product.

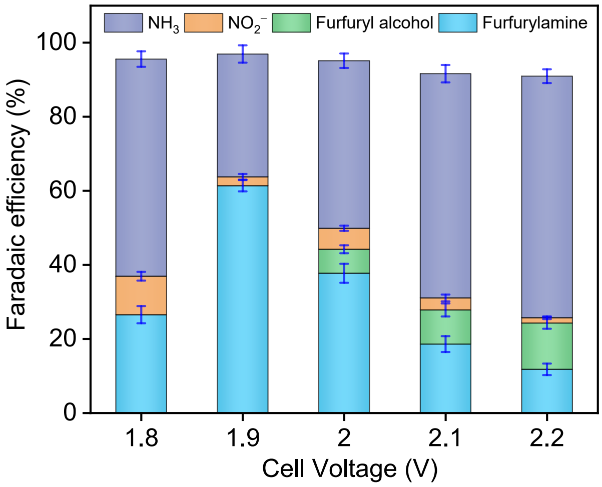

Fig. S60. Faradaic efficiencies of various products over HD-Cu-SA at different potentials in the SPCFR system.

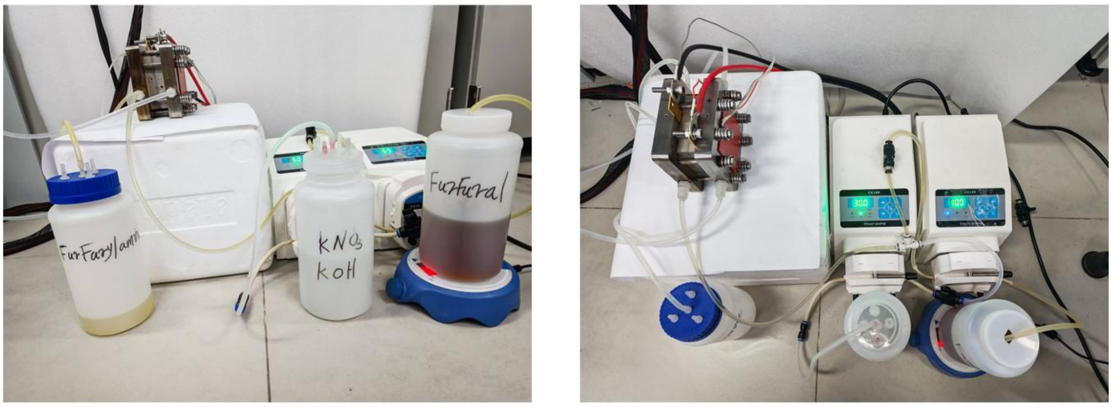

Fig. S61. The side view and top view of SPCFR system to synthesize furfurylamine.

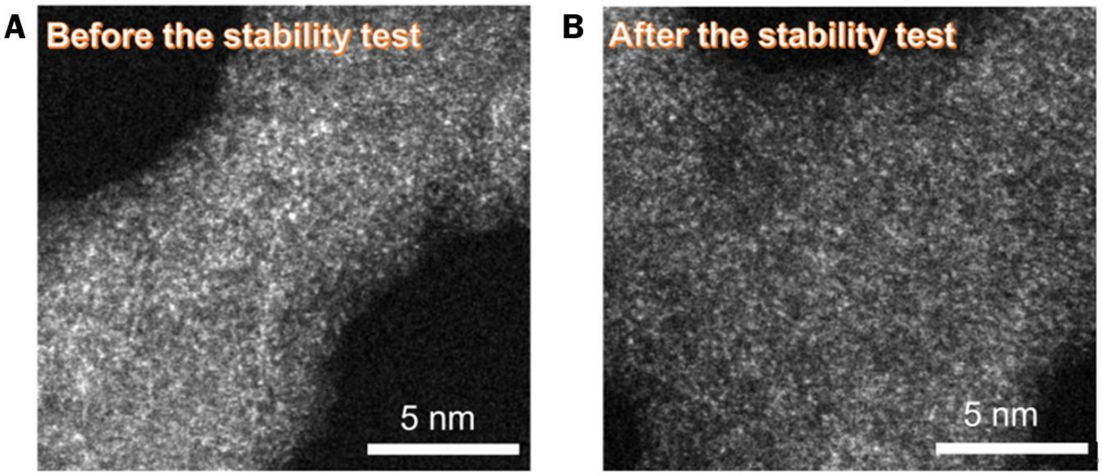

Fig. S62. HADDF-STEM images of HD-Cu-SA. **(A)** before the stability test and **(B)** after the stability test of 100 h co-reduction reaction in the SPCFR at a cell voltage of 1.9 V.

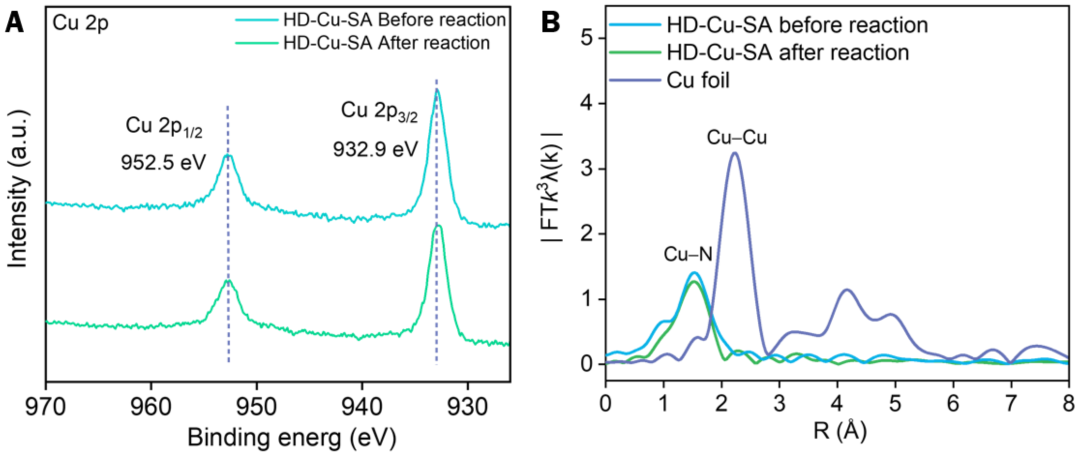

Fig. S63. XPS Spectra and EXAFS Spectra for Cu-SA. (A) Cu 2p XPS Spectra and **(B)** Cu K-edge EXAFS Spectra of HD-Cu-SA before and after the 100 h co-reduction reaction in the SPCFR at a cell voltage of 1.9 V.

Table S1 The content of Cu in various Cu-based catalysts determined by ICP-OES.

Samples	Cu (wt%)
Cu-SA	1.15
Cu-SNC	5.74
Cu-NPs	24.68
HD-Cu-SA	19.96

Table S2 The fitting results of EXAFS for Cu-SA.

Sample	Shell	N^a	R(Å)^b	σ^2(Å^2)^c	ΔE_0 (eV)^d	R factor
Cu foil	Cu-Cu	12*	2.54±0.01	0.0086	4.9±0.7	0.0046
Cu-SA	Cu-N	3.7±0.2	1.93±0.01	0.0083	1.0±1.7	0.0034

$^a N$: coordination numbers; $^b R$: bond distance; $^c \sigma^2$: Debye-Waller factors; ^{d} ΔE_0: the inner potential correction. R factor: goodness of fit.

Table S3 The list of control experiments

Entry	C-source	N-source	E (V vs. RHE)	Furfurylamine
1	Furfural	NO_3^-	−0.2 V	√
2	Furfural	×	−0.2 V	×
3	×	NO_3^-	−0.2 V	×
4	Furfural	NO_3^-	×	×
5	Furfural	NO_2^-	−0.2 V	√
6	Furfural	NH_2OH	−0.2 V	√
7	Furfural	NH_4^+	−0.2 V	×
8	Furfuryl alcohol	NO_3^-	−0.2 V	×
9	Furoic acid	NO_3^-	−0.2 V	×

Table S4 The yield rate and Faradaic efficiency of co-reduction various aldehyde and NO_3^-

Entry	Substrates	Products	Yeild rate μmol h^{-1} cm^{-2}	Faradaic efficiency
1	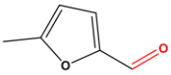	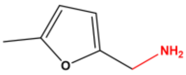	72.8	67.62 %
2	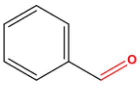	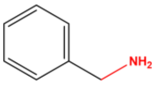	50.56	51.31 %
3	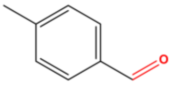	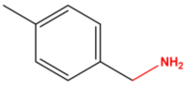	38.93	44.05 %
4	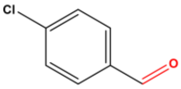	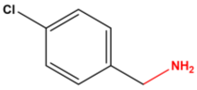	61.08	56.94 %
5	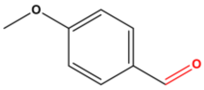	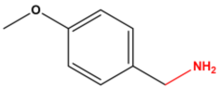	23.07	34.18 %
6	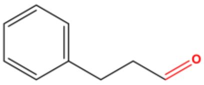	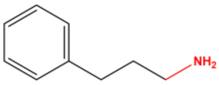	55.18	63.57 %

All primary amines are obtained by co-reduction of 0.045 M NO_3^- and 0.015 M aldehyde at −0.2 V vs. RHE

Table S5 Cu content of HD-Cu-SA before and after the stability test for 100 h at a cell voltage of 1.9 V in the SPCFR (determined by ICP-MS)

Samples	Cu (wt%)
HD-Cu-SA (Before the stability test)	19.96
HD-Cu-SA (After the stability test)	18.59

REFERENCES

1. T. Irrgang, R. Kempe, Transition-metal-catalyzed reductive amination employing hydrogen. *Chem. Rev.* **120**, 9583–9674 (2020).

2. K. Murugesan, T. Senthamarai, V. G. Chandrashekhar, K. Natte, P. C. J. Kamer, M. Beller, R. V. Jagadeesh, Catalytic reductive aminations using molecular hydrogen for synthesis of different kinds of amines. *Chem. Soc. Rev.* **49**, 6273–6328 (2020).

3. G. Liang, A. Wang, L. Li, G. Xu, N. Yan, T. Zhang, Production of primary amines by reductive amination of biomass-derived aldehydes/ketones. *Angew. Chem. Int. Ed. Engl.* **56**, 3050–3054 (2017).

4. T. Komanoya, T. Kinemura, Y. Kita, K. Kamata, M. Hara, Electronic effect of ruthenium nanoparticles on efficient reductive amination of carbonyl compounds. *J. Am. Chem. Soc.* **139**, 11493–11499 (2017).

5. J. Li, S. Gong, S. Gao, J. Chen, W.-W. Chen, B. Zhao, Asymmetric α-C(sp^3)−H allylic alkylation of primary alkylamines by synergistic Ir/ketone catalysis. *Nat. Commun.* **15**, 939 (2024).

6. Y. Zhang, L. Li, F. Liu, H. Qi, L. Zhang, W. Guan, Y. Liu, A. Wang, T. Zhang, Synergy between Ru and WO_x enables efficient hydrodeoxygenation of primary amides to amines. *ACS Catal.* **12**, 6302–6312 (2022).

7. G. Hahn, P. Kunnas, N. de Jonge, R. Kempe, General synthesis of primary amines via reductive amination employing a reusable nickel catalyst. *Nat. Catal.* **2**, 71–77 (2019).

8. X. Q. Ng, C. S. Lim, M. W. Liaw, T. T. Quach, B.-M. Yang, V. Isoni, J. Wu, Y. Zhao, Direct access to chiral aliphatic amines by catalytic enantioconvergent redox-neutral amination of alcohols. *Nat. Synth.* **2**, 572–580 (2023).

9. K. Lee, Y. Jing, Y. Wang, N. Yan, A unified view on catalytic conversion of biomass and waste plastics. *Nat. Rev. Chem.* **6**, 635–652 (2022).

10. H. Zhou, Y. Ren, B. Yao, Z. Li, M. Xu, L. Ma, X. Kong, L. Zheng, M. Shao, H. Duan, Scalable electrosynthesis of commodity chemicals from biomass by suppressing non-Faradaic transformations. *Nat. Commun.* **14**, 5621 (2023).

11. G. T. Jaya, R. Insyani, J. Park, A. F. Barus, M. G. Sibi, V. Ranaware, D. Verma, J. Kim, One-pot conversion of lignocellulosic biomass to ketones and aromatics over a multifunctional Cu–Ru/ZSM-5 catalyst. *Appl. Catal. Environ.* **312**, 121368 (2022).

12. P. Giri, S. Lim, T. P. Khobragade, A. D. Pagar, M. D. Patil, S. Sarak, H. Jeon, S. Joo, Y. Goh, S. Jung, Y.-J. Jang, S. B. Choi, Y. C. Kim, T. J. Kang, Y.-S. Heo, H. Yun, Biocatalysis enables the scalable conversion of biobased furans into various furfurylamines. *Nat. Commun.* **15**, 6371 (2024).

13. H. Zou, J. Chen, Efficient and selective approach to biomass-based amine by reductive amination of furfural using Ru catalyst. *Appl. Catal. Environ.* **309**, 121262 (2022).

14. J. Yu, G. Zhang, Y. Zeng, X. Ma, D. Lai, J. Zhang, Y. Zhang, T. Wang, Z.-J. Zhao, P. Zhang, J. Gong, Selective electrochemical C–N coupling via synergetic chemical and electrochemical microenvironment regulation strategies. *J. Am. Chem. Soc.* **147**, 43594–43603 (2025).

15. Yogita, K. T. V. Rao, P. M. Kumar, N. Lingaiah, Cobalt nanoparticles embedded in a nitrogen-doped carbon matrix for reductive amination of biomass-derived furfural to furfurylamine. *Sustain. Energy Fuels* **6**, 4692–4705 (2022).

16. S. Nishimura, K. Mizuhori, K. Ebitani, Reductive amination of furfural toward furfurylamine with aqueous ammonia under hydrogen over Ru-supported catalyst. *Res. Chem. Intermed.* **42**, 19–30 (2016).

17. H. Qi, J. Yang, F. Liu, L. Zhang, J. Yang, X. Liu, L. Li, Y. Su, Y. Liu, R. Hao, A. Wang, T. Zhang, Highly selective and robust single-atom catalyst Ru_1/NC for reductive amination of aldehydes/ketones. *Nat. Commun.* **12**, 3295 (2021).

18. Z. Xue, S. Wu, Y. Fu, L. Luo, M. Li, Z. Li, M. Shao, L. Zheng, M. Xu, H. Duan, Efficient light-driven reductive amination of furfural to furfurylamine over ruthenium-cluster catalyst. *J. Energy Chem.* **76**, 239–248 (2023).

19. J.-Y. Chen, Y. Xiao, F.-S. Guo, K.-M. Li, Y.-B. Huang, Q. Lu, Single-atom metal catalysts for catalytic chemical conversion of biomass to chemicals and fuels. *ACS Catal.* **14**, 5198–5226 (2024).

20. C. Liu, Y. Gao, B. Zhang, Organonitrogen electrosynthesis from CO_2 and nitrogenous sources in water. *Nat. Synth.* **3**, 794–796 (2024).

21. J. H. Jang, C. Kim, O. S. Nayal, J. B. Yeo, G. R. Kim, J. Kim, Y. I. Jo, U. Lee, M. S. Kwon, K. T. Nam, Electrochemically initiated synthesis of ethylene carbonate from CO_2. *Nat. Synth.* **3**, 846–857 (2024).

22. J. Li, H. Al-Mahayni, D. Chartrand, A. Seifitokaldani, N. Kornienko, Electrochemical formation of C–S bonds from CO_2 and small-molecule sulfur species. *Nat. Synth.* **2**, 757–765 (2023).

23. Y. Yuan, L. Chen, Z. Wan, K. Shi, X. Teng, H. Xu, P. Wu, J. Shi, Electrocatalytic ORR–coupled ammoximation for efficient oxime synthesis. *Sci. Adv.* **10**, eado1755 (2024).

24. S. Han, H. Li, T. Li, F. Chen, R. Yang, Y. Yu, B. Zhang, Ultralow overpotential nitrate reduction to ammonia via a three-step relay mechanism. *Nat. Catal.* **6**, 402–414 (2023).

25. J.-Y. Fang, Q.-Z. Zheng, Y.-Y. Lou, K.-M. Zhao, S.-N. Hu, G. Li, O. Akdim, X.-Y. Huang, S.-G. Sun, Ampere-level current density ammonia electrochemical synthesis using CuCo nanosheets simulating nitrite reductase bifunctional nature. *Nat. Commun.* **13**, 7899 (2022).

26. J. Wang, J. Cai, K.-X. Ren, L. Liu, S.-J. Zheng, Z.-Y. Wang, S.-Q. Zang, Stepwise structural evolution toward robust carboranealkynyl-protected copper nanocluster catalysts for nitrate electroreduction. *Sci. Adv.* **10**, eadn7556 (2024).

27. Q. Gao, B. Yao, H. S. Pillai, W. Zang, X. Han, Y. Liu, S.-W. Yu, Z. Yan, B. Min, S. Zhang, H. Zhou, L. Ma, H. Xin, Q. He, H. Zhu, Synthesis of core/shell nanocrystals with ordered

intermetallic single-atom alloy layers for nitrate electroreduction to ammonia. *Nat. Synth.* **2**, 624–634 (2023).

28. Y. Wu, Z. Jiang, Z. Lin, Y. Liang, H. Wang, Direct electrosynthesis of methylamine from carbon dioxide and nitrate. *Nat. Sustain.* **4**, 725–730 (2021).

29. W. Chen, Y. Wu, Y. Jiang, G. Yang, Y. Li, L. Xu, M. Yang, B. Wu, Y. Pan, Y. Xu, Q. Liu, C. Chen, F. Peng, S. Wang, Y. Zou, Catalyst selection over an electrochemical reductive coupling reaction toward direct electrosynthesis of Oxime from NOx and aldehyde. *J. Am. Chem. Soc.* **146**, 6294–6306 (2024).

30. S. Kuang, T. Xiao, H. Chi, J. Liu, C. Mu, H. Liu, S. Wang, Y. Yu, T. J. Meyer, S. Zhang, X. Ma, Acetamide electrosynthesis from CO_2 and Nitrite in Water. *Angew. Chem. Int. Ed. Engl.* **63**, e202316772 (2024).

31. C. Guo, W. Zhou, X. Lan, Y. Wang, T. Li, S. Han, Y. Yu, B. Zhang, Electrochemical upgrading of formic acid to formamide via coupling nitrite Co-reduction. *J. Am. Chem. Soc.* **144**, 16006–16011 (2022).

32. X. Huang, X. Li, S. Yan, D. Wang, C. Long, Y. Ying, P. An, Z. Guo, Q. Li, C. Yang, S. Chen, J. Han, L. Chang, S. Lu, Z. Tang, Strain-optimized copper dual-atom sites for selective electroreduction of carbon dioxide to ethylene. *Sci. Adv.* **11**, eads0609 (2025).

33. X. Chen, Y. Cheng, B. Zhang, J. Zhou, S. He, Gradient-concentration RuCo electrocatalyst for efficient and stable electroreduction of nitrate into ammonia. *Nat. Commun.* **15**, 6278 (2024).

34. X. Wang, C. Xu, M. Jaroniec, Y. Zheng, S.-Z. Qiao, Anomalous hydrogen evolution behavior in high-pH environment induced by locally generated hydronium ions. *Nat. Commun.* **10**, 4876 (2019).

35. C. E. Creissen, M. Fontecave, Keeping sight of copper in single-atom catalysts for electrochemical carbon dioxide reduction. *Nat. Commun.* **13**, 2280 (2022).

36. Q. Hu, W. Zhou, S. Qi, Q. Huo, X. Li, M. Lv, X. Chen, C. Feng, J. Yu, X. Chai, H. Yang, C. He, Pulsed co-electrolysis of carbon dioxide and nitrate for sustainable urea synthesis. *Nat. Sustain.* **7**, 442–451 (2024).

37. D. Zhang, Y. Xue, X. Zheng, C. Zhang, Y. Li, Multi-heterointerfaces for selective and efficient urea production. *Nat. Sci. Rev.* **10**, nwac209 (2023).

38. Y. Liu, Z. Zhuang, Y. Liu, N. Liu, Y. Li, Y. Cheng, J. Yu, R. Yu, D. Wang, H. Li, Shear-strained Pd single-atom electrocatalysts for nitrate reduction to ammonia. *Angew. Chem. Int. Ed. Engl.* **63**, e202411396 (2024).

39. R. Zhao, Y. Wang, J. Fu, F. Zhang, L. Wen, Y. Zhao, B. Guan, B. Han, Z. Liu, Achieving over 90% Faradaic efficiency in cyclohexanone oxime electrosynthesis using the Cu–Mo dual-site catalyst. *J. Am. Chem. Soc.* **146**, 27956–27963 (2024).

40. S. K. Nabil, M. A. Muzibur Raghuman, K. Kannimuthu, M. Rashid, H. S. Shiran, M. G. Kibria, M. A. Khan, Acid–base chemistry and the economic implication of electrocatalytic carboxylate production in alkaline electrolytes. *Nat. Catal.* **7**, 330–337 (2024).

41. H. Ni, Y. Wang, K. Yao, L. Wang, J. Huang, Y. Xiao, H. Chen, B. Liu, C. Y. Yang, J. Zhao, Cyclical palmitoylation regulates TLR9 signalling and systemic autoimmunity in mice. *Nat. Commun.* **15**, 1 (2024).

42. C. Xia, Y. Qiu, Y. Xia, P. Zhu, G. King, X. Zhang, Z. Wu, J. Y. Kim, D. A. Cullen, D. Zheng, P. Li, M. Shakouri, E. Heredia, P. Cui, H. N. Alshareef, Y. Hu, H. Wang, General synthesis of single-atom catalysts with high metal loading using graphene quantum dots. *Nat. Chem.* **13**, 887–894 (2021).

43. G. Kresse, D. Joubert, From ultrasoft pseudopotentials to the projector augmented-wave method. *Phys. Rev. B* **59**, 1758–1775 (1999).

44. J. P. Perdew, K. Burke, M. Ernzerhof, Generalized gradient approximation made simple. *Phys. Rev. Lett.* **77**, 3865–3868 (1996).

45. G. Henkelman, B. P. Uberuaga, H. Jónsson, A climbing image nudged elastic band method for finding saddle points and minimum energy paths. *J. Chem. Phys.* **113**, 9901–9904 (2000).
